# Inhibition and evasion of neutrophil microbicidal responses by *Legionella longbeachae*

**DOI:** 10.1128/mbio.03274-24

**Published:** 2024-12-16

**Authors:** Hannah E. Hanford, Christopher T. D. Price, Silvia Uriarte, Yousef Abu Kwaik

**Affiliations:** 1 Department of Microbiology and Immunology, College of Medicine, University of Louisville, Louisville, Kentucky, USA; 2 Center for Predictive Medicine, College of Medicine, University of Louisville, Louisville, Kentucky, USA; St. Jude Children's Research Hospital, Memphis, Tennessee, USA

**Keywords:** granules, lysosomes, ROS, NADPH oxidase, azurphilic granules

## Abstract

*Legionella* species evade degradation and proliferate within alveolar macrophages as an essential step for the manifestation of disease. However, most intracellular bacterial pathogens are restricted in neutrophils, which are the first line of innate immune defense against invading pathogens. Bacterial degradation within neutrophils is mediated by the fusion of microbicidal granules to pathogen-containing phagosomes and the generation of reactive oxygen species (ROS) by the phagocyte NADPH oxidase complex. Here, we show that human neutrophils fail to trigger microbicidal processes and, consequently, fail to restrict *L. longbeachae*. In addition, neutrophils infected with *L. longbeachae* fail to undergo a robust pro-inflammatory response, such as degranulation and IL-8 production. Here, we identify three strategies employed by *L. longbeachae* for evading restriction by neutrophils and inhibiting the neutrophil microbicidal response to other bacteria co-inhabiting in the same cell. First, *L. longbeachae* excludes the cytosolic and membrane-bound subunits of the phagocyte NADPH oxidase complex from its phagosomal membrane independent of the type 4 secretion system (T4SS). Consequently, infected neutrophils fail to generate robust ROS in response to *L. longbeachae*. Second, *L. longbeachae* impedes the fusion of azurophilic granules to its phagosome and the phagosomes of bacteria co-inhabiting the same cell through T4SS-independent mechanisms. Third, *L. longbeachae* protects phagosomes of co-inhabiting bacteria from degradation by ROS through a *trans*-acting T4SS-dependent mechanism. Collectively, we conclude that *L. longbeachae* evades restriction by human neutrophils *via* T4SS-independent mechanisms and utilizes *trans*-acting T4SS-dependent mechanisms for inhibition of neutrophil ROS generation throughout the cell cytosol.

**IMPORTANCE:**

*Legionella longbeachae* is commonly found in soil environments where it interacts with a wide variety of protist hosts and microbial competitors. Upon transmission to humans*, L. longbeachae* invades and replicates within alveolar macrophages, leading to the manifestation of pneumonia. In addition to alveolar macrophages, neutrophils are abundant immune cells acting as the first line of defense against invading pathogens. While most intracellular bacterial species are killed and degraded by neutrophils, we show that *L. longbeachae* evades degradation. The pathogen impairs the major neutrophils’ microbicidal processes, including the fusion of microbicidal granules to the pathogen-containing vacuole. By inhibiting of assembly of the phagocyte NADPH oxidase complex, the pathogen blocks neutrophils from generating microbicide reactive oxygen species. Overall, *L. longbeachae* employs unique virulence strategies to evade the major microbicidal processes of neutrophils.

## INTRODUCTION

*Legionella longbeachae* is a Gram-negative rod-shaped bacteria belonging to the *Legionellaceae* family that is occasionally found in water sources and ubiquitously found throughout soil environments (1–4). While amoeba and protists are the natural environmental hosts for *Legionella* species, humans serve as accidental hosts for *Legionella* following inhalation of contaminated environmental aerosols (3, 5–7). *Legionella* species are causative agents for Legionnaires’ disease, an atypical pneumonia that can be fatal without proper treatment (1, 6, 8). While *L. pneumophila* is responsible for roughly 90% of Legionnaires’ disease cases worldwide, *L. longbeachae* is the second most prevalent agent of Legionnaires’ disease and responsible for nearly two-thirds of cases reported in New Zealand and Australia (2, 5, 9). Interestingly, underlying health risks such as smoking or immunosuppression are less apparent in patients seeking medical care for *L. longbeachae* infections compared to *L. pneumophila*, suggesting *L. longbeachae* is more pathogenic (8–12). *In vivo* studies with murine models of Legionnaires’ disease support these case findings, where *L. longbeachae* is significantly more virulent and fatal during infection when compared to *L. pneumophila* (13–16).

Pathogenesis of both *L. longbeachae* and *L. pneumophila* is dependent on a functional Dot/Icm type IV secretion system (T4SS), which serves as a molecular syringe injecting a variety of protein effectors into the host cell cytosol to interfere with host response and promote bacterial replication (17–19). This injection of effector proteins promotes pathogen evasion of lysosomal degradation within the natural amoeba host and macrophages (17, 20–22). Interestingly, the T4SS effector substrates highly differ between *L. longbeachae* and *L. pneumophila* (1, 2, 17, 23–25). *L. longbeachae* reportedly harbors ~220 identified T4SS protein effectors compared to *L. pneumophila*, with at least 350 T4SS effectors known to date (1, 24, 25). Interestingly, *L. longbeachae* shares only ~30% effector homology with *L. pneumophila* and harbors ~51 unique effector proteins among *Legionella* species (1, 22, 24, 26). Although both species evade lysosomal fusion in macrophages, the intracellular trafficking of *L. longbeachae* is distinct from *L. pneumophila*. The *L. pneumophila-*containing vacuole excludes the LAMP-1 and LAMP-2 late endosomal markers as well as the lysosomal acid protease cathepsin D (27). By contrast, the *L. longbeachae*-containing vacuole acquires early endosomal antigen 1 (EEA1) along with the late endosomal LAMP-2 and mannose 6-phosphate receptor (M6PR) while excluding the Vacuolar ATPase and Cathepsin D lysosomal marker (27).

In addition to alveolar macrophages, neutrophils are abundantly present in pulmonary alveoli biopsies from patients and animal models infected with *L. longbeachae* or *L. pneumophila* (13, 28, 29). Accounting for at least 50% of peripheral blood leukocytes in humans, neutrophils are the first line of innate immune defense against microbial pathogens. Consequently, a dysregulation of the neutrophil presence and pro-inflammatory response can lead to devastating consequences (30–33). For example, the observed accumulation of neutrophils in host lungs during bacterial infection, while important for the restriction of invading pathogens, is considered to be one of the main contributors to host lung injury and respiratory distress during pneumonia progression (33, 34). The robust production of reactive oxygen species (ROS) and TNF signaling by neutrophils promotes the recruitment of immune cells to the lungs and enhances the pro-inflammatory response for clearance of *L. pneumophila* (35).

The mechanism for rapid microbicidal responses of human neutrophils that restrict *L. pneumophila* is mediated by the T4SS-injected *Legionella* amylase (LamA) effector into the cell cytosol, which triggers immuno-metabolic dysregulation and robust activation of infected neutrophils (36). The two major microbicidal mechanisms activated by neutrophils for restriction of *L. pneumophila* are the fusion of specific and azurophilic granules to the *L. pneumophila*-containing phagosome (*Lp*-LCP) and generation of intravacuolar ROS *via* assembly of the phagocyte NADPH oxidase complex on the *Lp*-LCP membrane (36, 37). However, *L. longbeachae* lacks the LamA effector protein (1, 13, 17, 36), and the intracellular fate of *L. longbeachae* within human neutrophils remains unknown.

Here, we show *L. longbeachae* evades degradation by human neutrophils independent of its T4SS. Neutrophils infected with *L. longbeachae* fail to recruit membrane-bound and cytosolic components of the NADPH oxidase complex to the *L. longbeachae*-containing phagosome (*Lo*-LCP) membrane, consequently failing to generate ROS. In addition, the *Lo*-LCP evades the fusion of azurophilic granules to the phagosomal membrane. Interestingly, *L. longbeachae* also protects co-inhabiting bacteria from the fusion of azurophilic granules and ROS production by T4SS-independent and T4SS-dependent strategies, respectively. Thus, *L. longbeachae* is one of few bacterial pathogens that evade degradation by human neutrophils by employing multiple strategies for inhibition of granule fusion and assembly of the catalytically active NADPH oxidase complex.

## RESULTS

### *L. longbeachae* evades degradation by neutrophils

**Fig 1:**
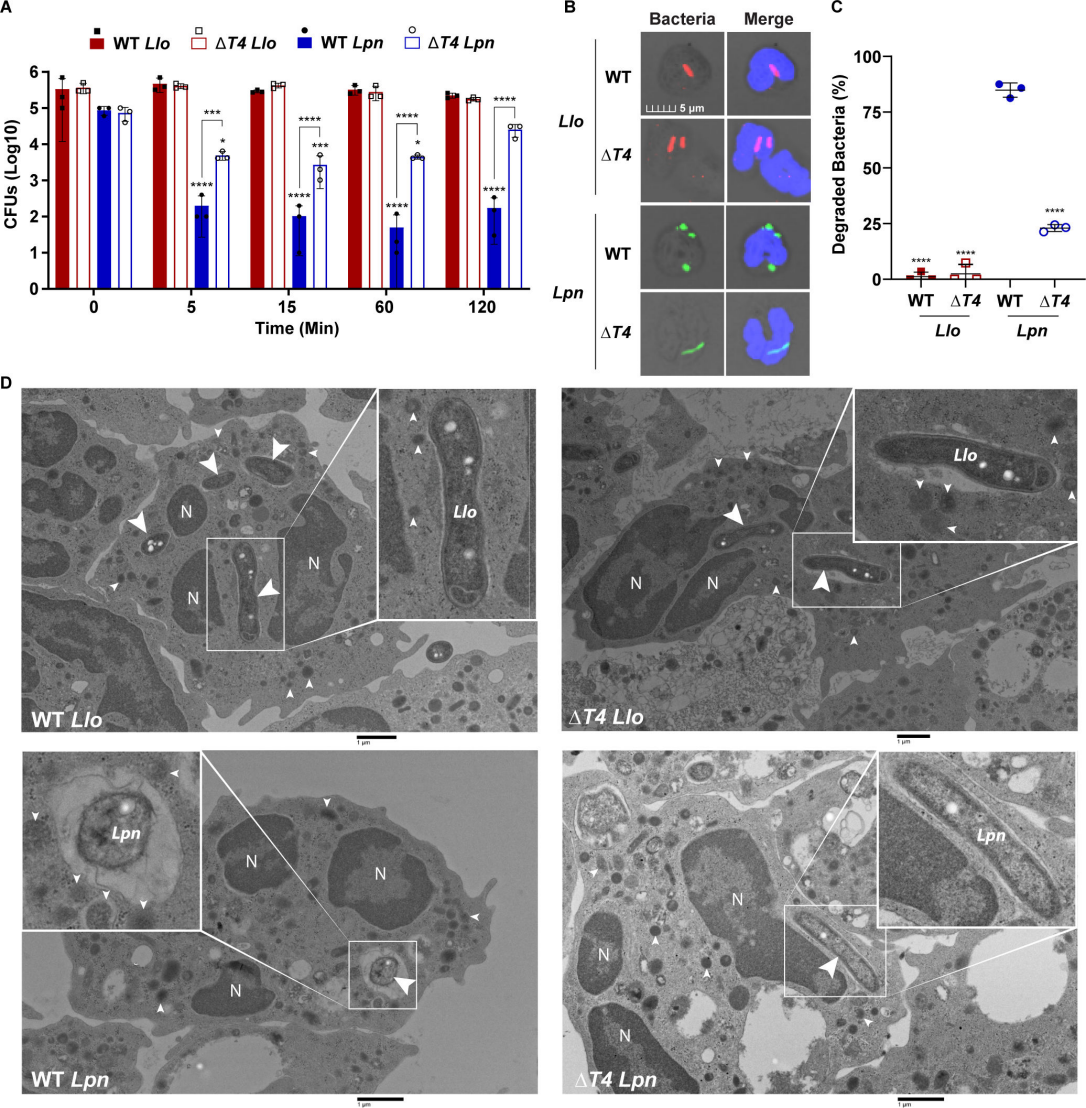
Intracellular survival of *L. longbeachae* in neutrophils. (**A**) To determine bacterial survival, infected neutrophils were lysed at 5, 15, 60, and 120 min post-infection. Serial dilutions of lysates were plated on BCYE agar for quantification of CFUs. ± SD, *n* = 3. (**B**) Representative confocal images of neutrophils infected with WT *L. longbeachae* (*Llo*), Δ*T4 Llo*, WT *L. pneumophila* (*Lpn*), or Δ*T4 Lpn* at 15 min post-infection. Bacteria were labeled with anti*-Llo* (red) or anti-*Lpn* (green) antibodies. Cell nuclei were stained with DAPI (blue). (**C**) Data are shown as mean percent killed and degraded *Llo* (red) or *Lpn* (blue) ±SD, *n* = 3 (scatter plot dots). (**D**) Representative TEM images of neutrophils infected for 1 h. Large white arrowheads indicate *Llo* or *Lpn*. Small white arrowheads indicate neutrophil granules. The data shown are representative of three independent biological repeats.

Little is known regarding human neutrophil response to *L. longbeachae*, the role of the T4SS during infection o, and intracellular fate of the pathogen. Since it was previously shown that opsonization of *L. pneumophila* was required for uptake and rapid degradation by human neutrophils at 15 min post-infection (36), we assessed whether opsonization with human serum was required for phagocytosis of *L. longbeachae* by neutrophils. Uptake of un-opsonized and serum-opsonized *L longbeachae* was determined at 15 min post*-*infection by differential labeling for intracellular and extracellular bacteria followed by assessment and 3D imaging with confocal microscopy (Fig. S1A). Opsonization of wild-type (WT) or *T4SS*-deficient (Δ*T4) L. longbeachae* with human serum resulted in a 40% and ~33% increase in bacterial uptake by neutrophils compared to un-opsonized bacteria, respectively (Student’s *t-*test*, P* < 0.0287, *P* > 0.05) (Fig. S1B). To determine the survival of *L. longbeachae* during infection of human neutrophils, neutrophils were lysed at 5, 15, 60, and 120 min post*-*infection. Total colony-forming units (CFU) for opsonized WT and Δ*T4 L. longbeachae* and its were determined. The WT and Δ*T4 L. pneumophila* were used as negative and positive controls for survival, respectively (36, 37). The data revealed the WT *L. pneumophila* control exhibited a ~ 1,000-fold decrease in bacterial viability as early as 15 min post-infection (ANOVA, *P* < 0.0001) (Fig. 1A) (36, 37). Interestingly, WT and Δ*T4 L. longbeachae* did not exhibit any significant changes in survival throughout 5, 15, 60, and 120 min post*-*infection (ANOVA, *P* > 0.05), whereas the Δ*T4 L. pneumophila* control still showed up to a 10-fold decrease in bacterial viability (ANOVA, *P* < 0.0289, *P* < 0.0006, *P* < 0.0232) (Fig. 1A).

To assess changes in bacterial morphology indicative of degradation, neutrophils were infected for 15 min with bacteria and examined by 3D imaging with confocal microscopy. Assessment of WT and Δ*T4 L. longbeachae* within neutrophils revealed normal rod-shaped morphology with less than 7% of bacteria showing rounded and amorphous morphology indicative of degradation and killing (Fig. 1B and C; Fig. S1C). As expected for the controls, ~82% of the WT *L. pneumophila* control exhibited rapid rounding and amorphous bacterial morphology at 15 min post-infection, while the Δ*T4 L. pneumophila* control maintained normal rod-shaped morphology with only 25% of bacteria killed and degraded (Fig. 1B and C) (36, 37). In addition, the CFUs from infected cell lysates showed no significant difference between exponentially and post-exponentially grown WT or Δ*T4 L. longbeachae*, indicating that the survival of *L. longbeachae* during infection of neutrophils was independent of the bacterial growth phase used for infection (Fig. S1D).

Transmission electron microscopy (TEM) of infected neutrophils at 1 h post*-*infection showed normal rod-shaped morphology for WT and Δ*T4 L. longbeachae* in infected neutrophils (Fig. 1D). In contrast to *L. longbeachae*, the WT *L. pneumophila* control exhibited increased vacuolar luminal space around killed and degraded bacteria, while the Δ*T4 L. pneumophila* control maintained normal rod-shaped morphology (Fig. 1D). Interestingly, a paucity of granule fusion to WT and Δ*T4 Lo*-LCPs was noted in TEM images, as the WT *L. pneumophila* control demonstrated abundant granule localization and fusion to the WT *Lp*-LCP (Fig. 1D inset, small white arrowheads). Together, these results indicate that *L. longbeachae* evades degradation by neutrophils and, surprisingly, this evasion is independent of the pathogen T4SS.

### Evasion of neutrophil microbicidal activities by *L. longbeachae*

**Fig 2:**
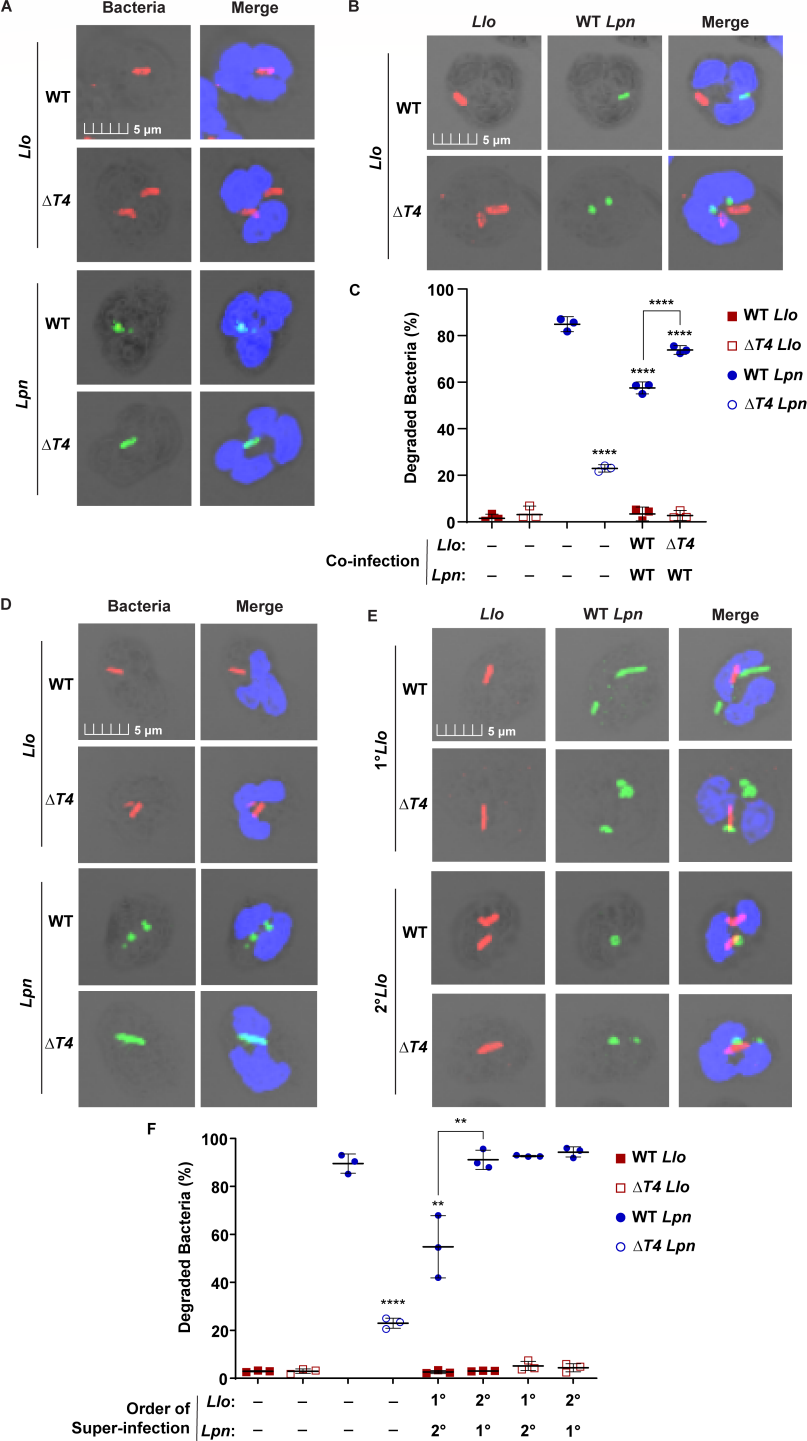
Trans-acting suppression of neutrophil microbicidal response by *L. longbeachae*. Quantification of killed and degraded *Llo* and *Lpn* during solo-infection or when co-inhabiting the same cell. (**A–C**) Neutrophils were solo-infected with WT or Δ*T4* bacterial strains for 15 min, or subjected to co-infections with *Llo* strains and WT *Lpn* at a multiplicity of infection (MOI) 1. Following a 15-min infection, bacterial morphology was examined by confocal microscopy in neutrophils solo-infected (**A**) or super-infected (**B**). (**C**) Data are shown as mean percent killed and degraded bacteria ± SD, *n* = 3 (scatter plot dots). (**D–F**) Neutrophils were solo-infected with WT or Δ*T4* bacterial strains for 15 min or subjected to 15-min primary infections (1°) followed by super-infection (2°) for an additional 15 min at an MOI of 1. Bacterial morphology was examined by confocal microscopy in neutrophils solo-infected (**D**) or super-infected (**E**). Bacteria were labeled with anti*-Llo* (red) or anti-*Lpn* (green) antibodies. Cell nuclei were stained with DAPI (blue). (**F**) Data are shown as mean percent killed and degraded *Llo* (red) or *Lpn* (blue) ±SD, *n* = 3 (scatter plot dots). The data shown are representative of three independent biological replicates.

Previous data showed that the rapid microbicidal response of human neutrophils to degrade *L. pneumophila* within 15 min is dependent on the T4SS (18, 36, 38–40). To determine whether neutrophils activated by the *L. pneumophila* T4SS can degrade *L. longbeachae*, cells were co-infected with both bacterial species for 15 min and examined by 3D imaging with confocal microscopy (Fig. 2A and B). The data showed that co-infection with *L. pneumophila* did not significantly increase degradation of WT or Δ*T4 L. longbeachae* compared to neutrophils infected with WT or Δ*T4 L. longbeachae* alone (solo-infected) (ANOVA, *P* > 0.05) (Fig. 2C). Remarkably, co-infected neutrophils exhibited a ~30% increase in survival of WT *L. pneumophila* compared to neutrophils solo-infected with the WT *L. pneumophila* control (ANOVA, *P* < 0.0001) (Fig. 2C). While co-infection with the Δ*T4 L. longbeachae* strain still resulted in a modest 10% increase in survival of WT *L. pneumophila,* the observed protection was significantly less than co-infection with WT *L. longbeachae* (ANOVA, *P* < 0.0001) (Fig. 2B and C). To confirm that the observed protection of *L. pneumophila* during co-infection was not specific for the NSW150 strain of *L. longbeachae*, the D4968 strain of *L. longbeachae* clinical isolate was used in 15 min co-infections and examined by 3D imaging with confocal microscopy (Fig. S2A and B). Both the NSW150 and D4968 strains of *L. longbeachae* resulted in ~31% increased survival of *L. pneumophila* in co-infected neutrophils (ANOVA, *P* < 0.0001) (Fig. S2C). Thus, the observed protection of *L. pneumophila* during co-infections with *L. longbeachae* was independent of the clinical isolate strain of *L. longbeachae*.

To determine whether the response of co-infected neutrophils was temporal, super-infection experiments were performed and compared to 15-min solo-infection controls (Fig. S3A). Human neutrophils were first infected (primary infection) with either *L. pneumophila* or *L. longbeachae* strains for 15 min, followed by secondary infection with the other species for an additional 15 min (Fig. S3A). Solo- and super-infected cells were examined by 3D imaging with confocal microscopy, and bacteria co-inhabiting the same cell (dual-infected) were assessed for morphology and degradation (Fig. 2D and E). Following primary infection by WT *L. longbeachae*, the survival of WT *L. pneumophila* increased by ~35% compared to the 15-min solo-infected WT *L. pneumophila* control (ANOVA, *P* < 0.0057) (Fig. 2B). Importantly, primary infection by the Δ*T4* mutant of *L. longbeachae* failed to protect WT *L. pneumophila* from degradation during super-infections, confirming that the observed protection of *L. pneumophila* during *L. longbeachae* primary infection was largely T4SS dependent (ANOVA, *P* < 0.0049) (Fig. 2B). Super-infections were also performed with *L. longbeachae* strains and Δ*T4 L. pneumophila* (Fig. S3B through D). Primary infection with WT and Δ*T4 L. longbeachae* strains had no effect on the survival of Δ*T4 L. pneumophila* (ANOVA, *P* > 0.05) (Fig. S3C). In addition, primary infection with WT or Δ*T4 L. pneumophila* strains did not significantly impact the survival of WT or Δ*T4 L. longbeachae* compared to the WT or Δ*T4 L. longbeachae* 15-min solo-infection controls (ANOVA, *P* > 0.05) (Fig. 2B; Fig. S3D).

To determine whether there was a difference in bacterial uptake for solo- and dual-infected cells during super-infections, super-infected neutrophils were examined by confocal microscopy and the percent of *L. longbeachae* solo-infected cells, *L. pneumophila* solo-infected cells, and dual-infected cells were quantified relative to total cell counts (Fig. S4A and B). WT and Δ*T4 L. longbeachae* strains exhibited no significant differences in uptake by solo- or dual-infected cells across the super-infection groups, and the total percent of cells infected with WT or Δ*T4 L. longbeachae* across all super-infection groups was similar to the WT and Δ*T4 L. longbeachae* solo-infection controls (ANOVA, *P* > 0.05) (Fig. S4A). The total percent of cells infected with WT *L. pneumophila* across all super-infection groups was similar to the WT and Δ*T4 L. pneumophila* solo-infection controls (ANOVA, *P* > 0.05) (Fig. S4B).

To determine whether the observed protection of *L. pneumophila* was dependent on co-inhabiting the same cell with *L. longbeachae* during super-infections, bacteria within *L. longbeachae* solo-infected cells, *L. pneumophila* solo-infected cells, and dual-infected cells were quantified and examined for degraded morphology (Fig. S4C and D). WT and Δ*T4 L. longbeachae* exhibited no significant difference in survival between solo- or dual-infected cells in super-infection groups, and the total percent survival of WT and Δ*T4 L. longbeachae* was similar to bacteria from WT and Δ*T4 L. longbeachae* solo-infection controls (ANOVA, *P* > 0.05) (Fig. S4C). Interestingly, following primary infection with WT *L. longbeachae*, the WT *L. pneumophila* exhibited ~20% increase in bacterial survival in only dual-infected cells (ANOVA, *P* < 0.0014), while the WT *L. pneumophila* solo-infected cells during super-infection were not significantly different from the WT *L. pneumophila* solo-infection control (ANOVA, *P* > 0.05) (Fig. S4D). Following primary infection with Δ*T4 L. longbeachae*, the WT *L. pneumophila* exhibited a modest but significant ~10% increase in bacterial survival of dual-infected cells compared to the solo-infection WT *L. pneumophila* control (ANOVA, *P* < 0.0058) (Fig. S4D). The survival of WT *L. pneumophila* in dual-infected cells following primary infection with Δ*T4 L. longbeachae* was not significantly different from *L. pneumophila* solo-infected cells during super-infections (ANOVA, *P* > 0.05) (Fig. S4D). Therefore, while *L. longbeachae* exhibits a T4SS-independent evasion of degradation by neutrophils, there is largely T4SS-dependent *trans*-acting protection of co-inhabiting bacteria from degradation within dual-infected cells.

The CFUs were enumerated during co-infections to determine whether the protection of *L. pneumophila* by WT *L. longbeachae* could be detected without single-cell analysis (Fig. S5). To select for each *Legionella* species on BCYE plates, *L. longbeachae* strains were transformed with a kanamycin resistance plasmid and *L. pneumophila* was transformed with a chloramphenicol resistance plasmid. Co-infected cells were lysed at 0, 0.5, 1, 2, and 20 h post-infection and lysates were plated on kanamycin or chloramphenicol plates to select for *L. longbeachae* and *L. pneumophila*, respectively. The WT and Δ*T4 L. longbeachae* demonstrated no significant change in survival aside from a mild few-fold decrease by 1 h post-infection, but this decrease was inconsistent across three different experimental designs and nine total experimental repeats (Fig. S5A; Table S1). Enumeration of bacterial survival by CFUs showed no detectable protection of WT *L. pneumophila* by *L. longbeachae* strains during co-infection (Fig. S5A; Table S1). However, these data were inconsistent across several biological repeats due to variability in maintaining the antibiotic resistance plasmids during infection. The lack of detectable protection of WT *L. pneumophila* by *L. longbeachae* determined by CFU enumeration is likely due to only a minority of the cells (~20%) being dual-infected by both bacterial species. In addition, the number of viable bacteria within dual-infected vs total infected cells is likely too low to be detected by CFU enumeration since the protection was partial. Altogether, *L. longbeachae* evasion of degradation by human neutrophils is independent of the *L. longbeachae* growth phase and T4SS. In addition, the protection of *L. pneumophila* from degradation by human neutrophils is largely dependent on the *L. longbeachae* T4SS when co-inhabiting the same cell during single-cell analysis.

### Failure of fusion of azurophilic granules to the *L. longbeachae*-containing phagosomes

Neutrophil azurophilic granules contain cationic antimicrobial peptides including α-defensins, myeloperoxidase, proteinase 3, and elastase, while specific granules contain antimicrobial proteins such as cathelicidin, lactoferrin, and lysozyme (37, 41). As neutrophils undergo activation, these pre-made microbicidal granules located throughout the cell cytosol are mobilized and recruited for fusion to pathogen-containing phagosomes or to the plasma membrane for exocytosis into the environment (42). In our TEM imaging of neutrophils at 1 h post-infection, we noted a possible failure in granule fusion to the *Lo-*LCP compared to the *Lp*-LCP (Fig. 1D). Therefore, we determined whether *L. longbeachae* evasion of degradation by human neutrophils was mediated by subverting fusion of azurophilic and specific granules to the *Lo-*LCP. Neutrophils were stimulated with Zymosan A BioParticles as a positive control for the phagosomal fusion of azurophilic and specific granules.

**Fig 3:**
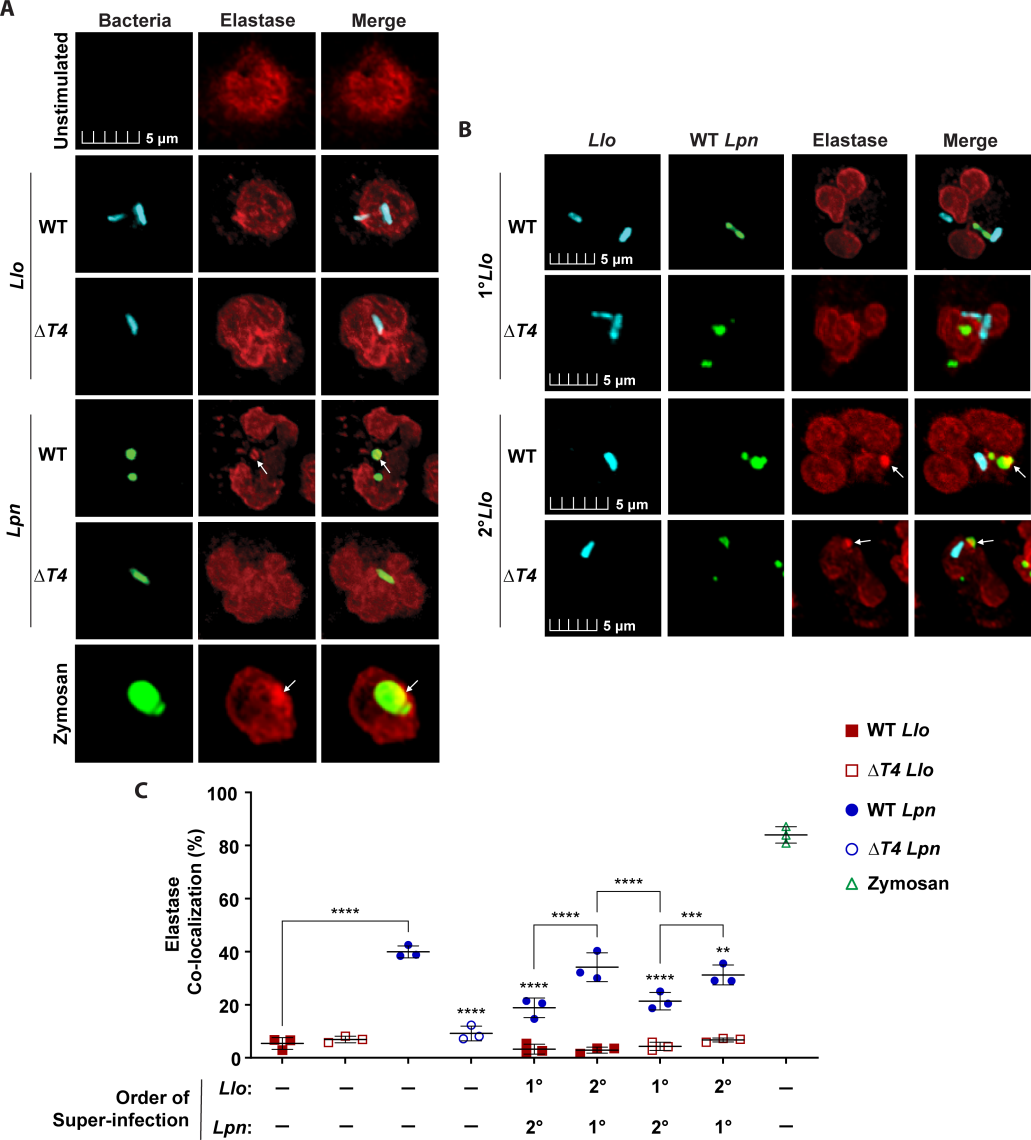
Inhibited fusion of azurophilic granules to the *L. longbeachae*-containing phagosome. To determine the fusion of azurophilic granules to bacterial phagosomes, neutrophils were labeled for elastase. Neutrophils were solo-infected with WT or Δ*T4* bacterial strains for 15 min or subjected to 15-min primary infections (1°) followed by super-infection (2°) for an additional 15 minutes. Representative confocal images of azurophilic granule fusion to phagosomes in neutrophils solo-infected (**A**) or super-infected (**B**). Bacteria were labeled with anti-*Llo* antibody (cyan) or anti-*Lpn* antibody (green). Zymosan A BioParticles Alexa Fluor 594 are shown in green. Azurophilic granules were labeled with anti-elastase antibodies (red). White arrows indicate positive co-localization of elastase with pathogen-containing phagosomes. (**C**) Data are shown as mean percent co-localization of elastase to *Llo* phagosomes (red) or *Lpn* phagosomes (blue) during infections. Zymosan was included as a positive control (green) ± SD, *n* = 3 (scatter plot dots). The data shown are representative of three independent biological repeats.

To determine the fusion of azurophilic granules to bacterial phagosomes, neutrophils were examined by confocal microscopy for detection of elastase at pathogen-containing phagosomes at 15 min post-infection, using specific antibodies (Table 1) (43). During the 15-min solo-infection of neutrophils with WT or Δ*T4 L. longbeachae*, less than 8% of *Lo*-LCPs showed co-localization with elastase (Fig. 3A and C). By contrast, neutrophils solo-infected with the WT *L. pneumophila* control exhibited 40% co-localization with elastase, while co-localization of elastase to the Δ*T4 L. pneumophila* control was less than 10% in comparison (ANOVA, *P* < 0.0001) (Fig. 3A and C). Therefore, the *Lo*-LCP demonstrates T4SS-independent evasion of azurophilic granule fusion.

**TABLE 1:** Antibodies used in this study

Antibody	Species	Experimental use	Dilution	Source
Anti-*L. pneumophila*	Rabbit	Intracellular bacterial morphology (solo- and co-infections), degradation super-infections, p47phox super-infections, NOX2 infections	1:1,000	YA Lab
Anti-*L. longbeachae*	Chicken	Intracellular bacterial morphology (solo- and co-infections), granule super-infections, p47phox super-infections, NOX2 infections	1:4,000	Aves Labs
Anti-*L. pneumophila*	Goat	Granule super-infections	1:1,000	GenScript
Anti-*L. longbeachae*	Rabbit	Uptake and degradation of solo-infections	1:5,000	CDC
Anti-lactoferrin	Rabbit	Specific granule super-infections	1:100	Abcam, #Ab109216
Anti-NGAL	Rabbit	Specific granule super-infections	1:100	ThermoFisher, #PA5-79589
Anti-CD66b	Rabbit	Specific granule super-infections	1:200	# PA5-104296
Anti-elastase	Mouse	Azurophilic granule super-infections	1:100	Abcam, #Ab254178
Anti-p47phox antibody	Goat	p47phox super-infections	1:100	Abcam, #Ab166930
Anti-NOX2 antibody	Rabbit	NOX2 infections	1:200	ThermoFisher, #PA5-141135
				
Anti-rabbit Alexa Fluor 405	Donkey	Granule super-infections	1:250	Invitrogen, #A48257
Anti-chicken Alexa Fluor 488	Donkey	NOX2 infections	1:2,000	Invitrogen, #A78948
Anti-mouse Alexa Fluor 488	Donkey	Granule super-infections	1:2,000	Invitrogen, #A32766
Anti-rabbit Alexa Fluor 488	Donkey	Intracellular bacterial morphology (solo- and co-infections), degradation super-infections	1:2,000	Invitrogen, #A32790
	Donkey	Intracellular bacterial morphology (solo- and co-infections), degradation super-infections	1:2,000	Invitrogen, #A78949
Anti-goat Alexa Fluor 555	Donkey	Granule super-infections	1:2,000	Invitrogen, #A21432
Anti-mouse Alexa Fluor Plus 555	Donkey	NOX2 infections	1:1,000	Invitrogen, #A32773
Anti-chicken Alexa Fluor 647	Donkey	Granule super-infections, p47phox super-infections	1:2,000	Invitrogen, #A78952
Anti-rabbit Alexa Fluor 647	Donkey	NOX2 infections	1:2,000	Invitrogen, #A31573
DAPI (405)		Intracellular bacterial morphology (solo- and co-infections), p47phox super-infections, NOX2 infections	1:5,000	Millipore Sigma, #80051–386

To determine whether *L. longbeachae* exhibited a *trans*-acting inhibition of azurophilic granule fusion to co-inhabiting bacteria, we performed super-infections and assessed elastase localization to pathogen-containing phagosomes, as described above. Interestingly, compared to the 15-min solo-infection by the WT *L. pneumophila* control, primary infection of neutrophils with WT or Δ*T4 L. longbeachae* protected *L. pneumophila* from azurophilic granule fusion*,* resulting in ~20% decreased co-localization of elastase with WT *Lp-*LCPs (ANOVA, *P* < 0.0001) (Fig. 3B and C). In addition, primary infection of neutrophils with WT *L. pneumophila* did not significantly increase elastase co-localization with WT or Δ*T4 Lo-*LCPs compared to the 15-min solo-infection control with WT or Δ*T4 L. longbeachae* (ANOVA, *P* > 0.05) (Fig. 3B and C). Thus, *L. longbeachae* employs a T4SS-independent *trans*-acting strategy for inhibiting the fusion of azurophilic granules to its own phagosome and phagosomes of *L. pneumophila* within dual-infected cells.

**Fig 4:**
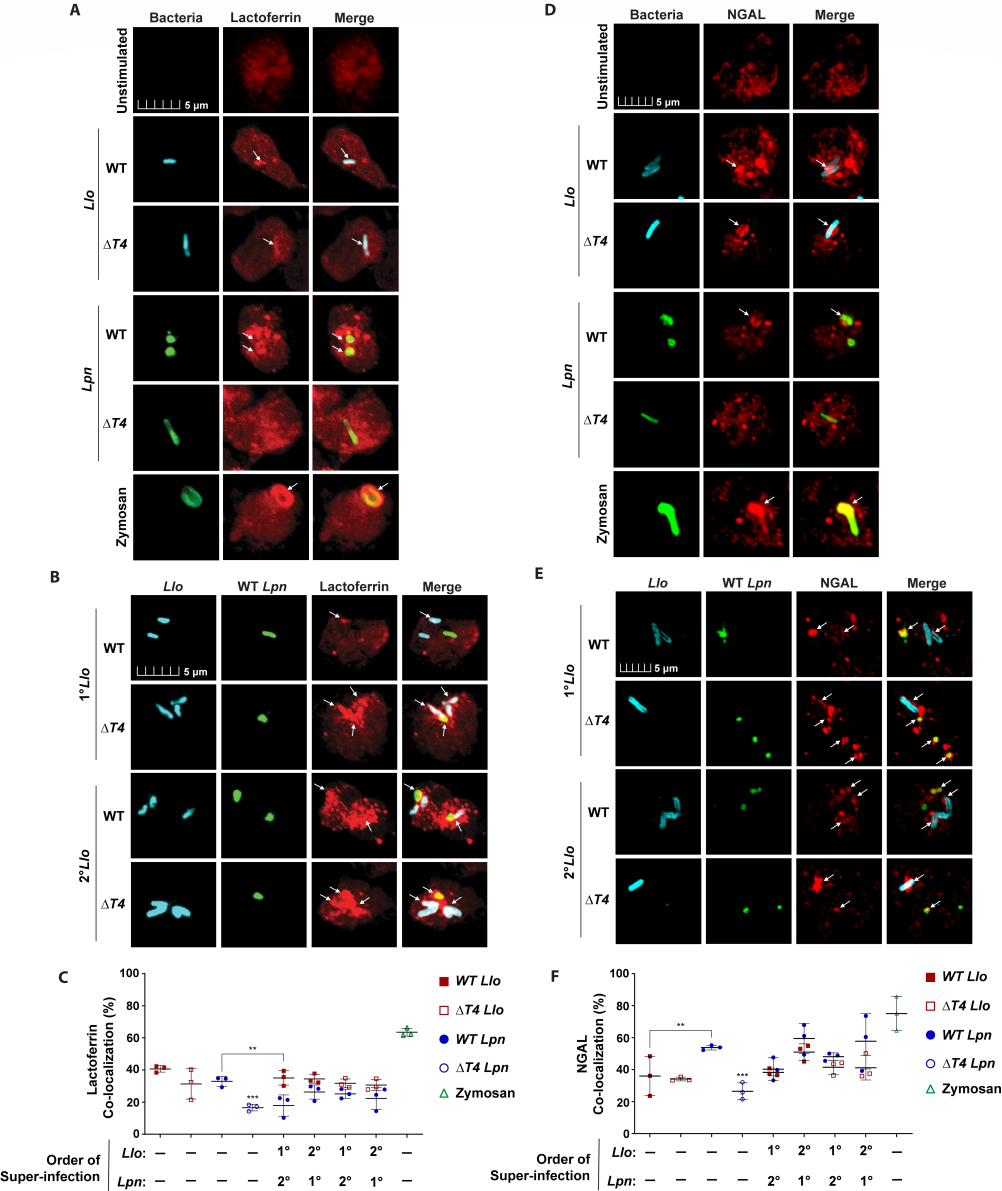
Fusion of specific granules to the *L. longbeachae*-containing phagosome. To determine the fusion of specific granules to bacterial phagosomes, neutrophils were labeled for lactoferrin (**A–C**) and NGAL (**D–F**). Neutrophils were solo-infected with WT or Δ*T4* bacterial strains for 15 min or subjected to 15 min primary infections (1°) followed by super-infection (2°) for an additional 15 minutes. Representative confocal images of specific granule fusion to phagosomes during solo-infections (**A and D**) or super-infections (**B and E**). Bacteria were labeled with anti-*Llo* antibody (cyan) or anti-*Lpn* antibody (green). Zymosan A BioParticles Alexa Fluor 594 are shown in green. Specific granules were labeled with anti-lactoferrin or anti-NGAL antibodies (red). White arrows indicate positive co-localization of lactoferrin or NGAL with pathogen-containing phagosomes. (**C and F**) Data are shown as mean percent co-localization of lactoferrin or NGAL to *Llo* phagosomes (red) or *Lpn* phagosomes (blue) during infections. Zymosan was included as a positive control (green) ± SD, *n* = 3 (scatter plot dots). The data shown are representative of three independent biological repeats.

Fusion of specific granules to phagosomes during 15-min solo-infections was determined by examining infected cells with confocal microscopy for detection of lactoferrin at pathogen-containing phagosomes using specific antibodies (Table 1) (44, 45). Solo-infection of neutrophils with WT or Δ*T4 L. longbeachae* resulted in 41% and 32% co-localization of lactoferrin to the *Lo*-LCPs at 15 min post*-*infection, respectively (Fig. 4A and C). In addition, neutrophils solo-infected with the WT or Δ*T4 L. pneumophila* controls exhibited ~33% and less than 19% co-localization of lactoferrin to *Lp*-LCPs, respectively (Fig. 4A and C). Interestingly, co-localization of lactoferrin to WT or Δ*T4 Lo*-LCPs during solo-infections was not significantly different from the WT *L. pneumophila* positive control (ANOVA, *P* > 0.05) (Fig. 4A and C), suggesting that *L. longbeachae* does not evade specific granule fusion to its own phagosome, and nor do specific granules degrade *L. longbeachae*. The data showed no significant change in lactoferrin co-localization to WT or Δ*T4 Lo-*LCPs compared to solo-infection *Lo*-LCPs following primary infection of WT *L. pneumophila* (ANOVA, *P* > 0.05) (Fig. 4B and C). In addition, primary infection with WT *L. longbeachae* only resulted in a mild reduction of lactoferrin co-localization to WT *Lp-*LCPs compared to the WT *L. pneumophila* solo-infection control (ANOVA, *P <* 0.0022), but this observed reduction of lactoferrin co-localization to WT *Lp-*LCPs was inconsistent across experimental repeats. Primary infection of neutrophils with Δ*T4 L. longbeachae* did not significantly reduce *Lp-*LCP co-localization with lactoferrin (ANOVA, *P* > 0.05) (Fig. 4B and C).

Since the data for the fusion of specific granules to *Lo*-LCPs were different than what was observed for azurophilic granules, the above experiments were repeated using another specific granule matrix marker (NGAL) (46, 47) and a membrane marker (CD66b) (Fig. 4D through F; Fig. S6) (48, 49). Solo-infection of neutrophils with WT or Δ*T4 L. longbeachae* resulted in ~38% co-localization of NGAL to the *Lo*-LCPs at 15 min post*-*infection, while neutrophils solo-infected with the WT or Δ*T4 L. pneumophila* controls exhibited 55% and 25% co-localization of NGAL to *Lp*-LCPs, respectively (Fig. 4D and F). Co-localization of NGAL to WT or Δ*T4 Lo*-LCPs during solo-infections was 18% less than the WT *L. pneumophila* positive control (ANOVA, *P* < 0.0095) (Fig. 4D and F), but this difference was inconsistent across experimental repeats (data not shown). Primary infection with WT or Δ*T4 L. longbeachae* did not change the co-localization of NGAL to WT *Lp*-LCPs compared to the WT *L. pneumophila* solo-infection control (ANOVA, *P* > 0.05) (Fig. 4E and F). Following primary infection of WT *L. pneumophila*, the data showed no significant difference in NGAL co-localization to WT or Δ*T4 Lo-*LCPs compared to solo-infection *Lo*-LCPs (ANOVA, *P* > 0.05) (Fig. 4E and F). Therefore, the *Lo*-LCP acquires the NGAL and lactoferrin markers for specific granules.

Contrary to the data for lactoferrin and NGAL, neutrophils solo-infected with WT or Δ*T4 L. longbeachae* exhibited less than 8% CD66b co-localization to WT and Δ*T4 Lo*-LCPs, while neutrophils solo-infected with WT or Δ*T4 L. pneumophila* exhibited 40% and 10% co-localization of CD66b to WT and Δ*T4 Lp*-LCPs, respectively (ANOVA, *P* < 0.0001) (Fig. S6A and C). Primary infection with WT or Δ*T4 L. longbeachae* resulted in no significant change in CD66b co-localization to WT *Lp-*LCPs compared to solo-infection *Lp*-LCPs that was consistent across experimental repeats (ANOVA, *P* > 0.05) (Fig. S6B and C). In addition, primary infection with WT *L. pneumophila* did not significantly increase CD66b co-localization to WT or Δ*T4 Lo*-LCPs (ANOVA, *P* > 0.05) (Fig. S6B and C). While CD66b data were significantly different from lactoferrin and NGAL, CD66b is often used as a marker for neutrophil activation by assessing expression on cell surfaces (50, 51). Thus, CD66b may be best suited for flow cytometry or degranulation analysis for cell activation as opposed to assessing intracellular fusion of specific granules to pathogen phagosomes.

Overall, the data show that *Lo*-LCPs acquire protein markers of specific granules, and *L. longbeachae* does not protect phagosomes of bacteria co-inhabiting dual-infected cells from fusion to specific granules. Therefore, the data suggest that specific granules do not play a detectable role in the restriction of *L. longbeachae.* This is consistent with the previous findings that specific granules do not play a detectable role in the restriction of *L. pneumophila* by human neutrophils (36).

### Inhibition of neutrophil ROS production by *L. longbeachae*

Since neutrophils produce intracellular ROS (icROS) for rapid degradation of phagocytosed bacteria*,* including *L. pneumophila* (36), we determined whether *L. longbeachae* evades degradation by neutrophils through inhibition of ROS production. Prior to ROS assays, the neutrophil response to formalin-killed, heat-killed, and UV-killed *L. longbeachae* was assessed at 1 h post-infection to determine a control for *L. longbeachae* that would induce PMN microbicidal responses (Fig. S7A through C). Neutrophil response to killed bacteria was determined by assessing bacterial uptake and degradation using confocal microscopy (Fig. S7A). There was no difference in uptake for viable or killed *L. longbeachae* strains (Fig. S7B). Since UV-killed *L. longbeachae* was rapidly degraded by neutrophils (Student’s *t-*test, *P* < 0.0001) (Fig. S7A and C), it was included as a control for kinetics of ROS generation in addition to WT *L pneumophila*.

**Fig 5:**
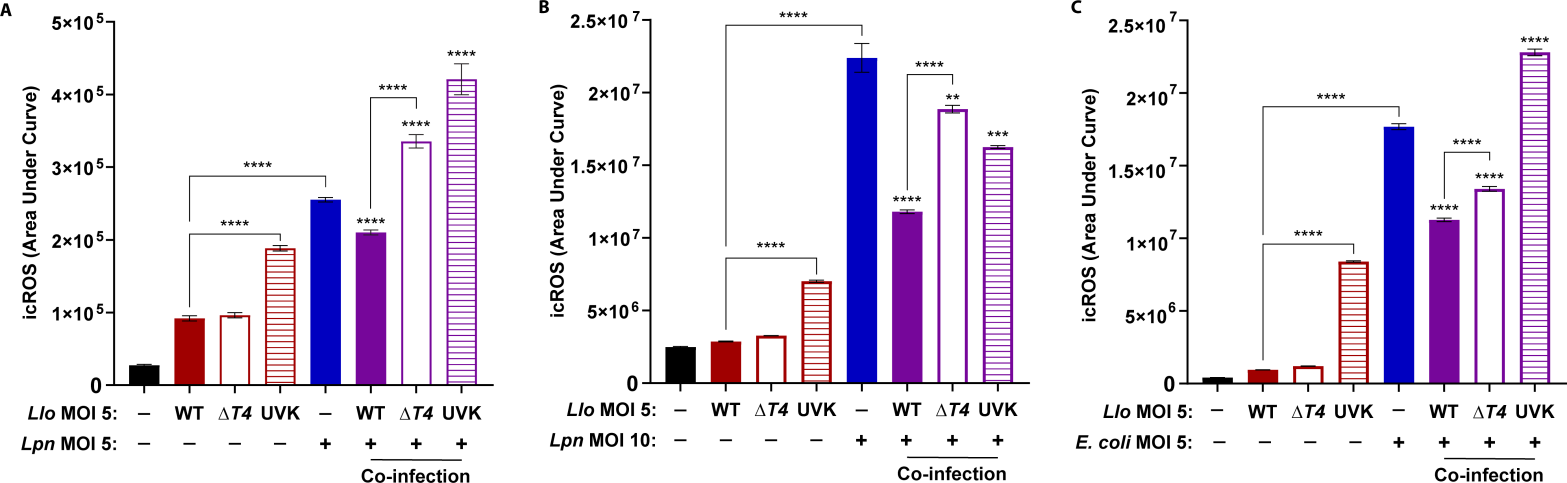
Failure of neutrophils to elicit robust ROS production in response to *L. longbeachae*. Neutrophils were infected with WT or Δ*T4 Legionella* strains alone, UV-killed (UVK) *Llo* alone, or co-infected as indicated on the x-axis. The kinetic of ROS production was determined up to 1 h. Neutrophils were solo-infected or co-infected with *Legionella* strains at a 1:1 infection ratio of *Llo* to *Lpn* with an MOI of 5 (**A**) or 1:2 infection ratio of *Llo* to *Lpn* (**B**) with an MOI of 5 and 10, respectively ±SD, *n* = 3. (**C**) Neutrophils were solo-infected or co-infected with *E. coli* and *Llo* strains at a 1:1 infection ratio with an MOI of 5. Data are shown as mean area under curve for kinetic ROS production during 1 h post-infection for unstimulated (black), *Llo*-infected (red), *Lpn*- or *E. coli*-infected (blue), and co-infected cells (purple) ±SD, *n* = 3. The data shown are representative of three independent biological repeats.

Neutrophils were solo- or co-infected at a multiplicity of infection (MOI) of 5 for each bacterial strain (Fig. 5A). Neutrophils solo-infected with viable WT or Δ*T4 L. longbeachae* did not significantly increase ROS generation compared to unstimulated cells (ANOVA, *P* > 0.05) (Fig. 5A). By contrast, infection with UV-killed *L. longbeachae* resulted in significant ROS generation similar to the WT *L. pneumophila* positive controls (ANOVA, *P* < 0.0001) (Fig. 5A). Therefore, failure in robust ROS generation by neutrophils in response to viable *L. longbeachae* was independent of the *L. longbeachae* T4SS. Compared to the *L. pneumophila* solo-infection control, co-infection of WT *L. longbeachae* with WT *L. pneumophila* exhibited a significant reduction of ROS production by neutrophils (ANOVA, *P* < 0.0001) (Fig. 5A). By contrast, co-infecting Δ*T4 L. longbeachae* or UV-killed *L. longbeachae* with *L. pneumophila* resulted in robust ROS generation (ANOVA, *P* < 0.0001) (Fig. 5A).

To determine whether *L. longbeachae* inhibition of ROS generation could be observed in the presence of a larger number of *L. pneumophila*, the above experiment was repeated while maintaining *L. longbeachae* strains at an MOI of 5 and increasing WT *L. pneumophila* to an MOI of 10 for a 1:2 infection ration (Fig. 5B). Interestingly, WT *L. longbeachae* still significantly inhibited neutrophil ROS production during co-infection with WT *L. pneumophila* at an MOI of 10 (ANOVA, *P* < 0.0001) (Fig. 5B). In addition, infecting neutrophils with *L. pneumophila* at an MOI of 10 increased the scale of ROS production and revealed a mild inhibition of ROS generation during co-infection with Δ*T4 L. longbeachae* (ANOVA, *P* < 0.0041) (Fig. 5B). However, ROS generation during co-infection of *L. pneumophila* with Δ*T4 L. longbeachae* was still significantly higher than co-infection of *L. pneumophila* with WT *L. longbeachae* (ANOVA, *P* < 0.0001) (Fig. 5B). Thus, *L. longbeachae* exhibits a largely T4SS-dependent *trans*-acting inhibition of ROS generation by neutrophils that protects co-infecting bacteria.

To determine whether WT *L. longbeachae* reduced neutrophil ROS production to other intracellular pathogens in addition to *L. pneumophila*, co-infections were repeated with *E. coli* at an MOI of 5. Co-infection of WT *L. longbeachae* with *E. coli* resulted in a significant decrease in ROS production compared to the *E. coli* solo-infection control (ANOVA, *P* < 0.0001) (Fig. 5C). In contrast to WT *L. longbeachae*, co-infection of UV-killed *L. longbeachae* with *E. coli* restored robust ROS generation by infected neutrophils (ANOVA, *P* < 0.0001) (Fig. 5C). Interestingly, the increased scale of ROS production in response to *E. coli* revealed a greater inhibition of ROS generation during co-infection with Δ*T4 L. longbeachae* (ANOVA, *P* < 0.0001) (Fig. 5C). However, ROS generation for co-infection of *E. coli* with Δ*T4 L. longbeachae* was still significantly higher than co-infection of *E. coli* with WT *L. longbeachae* (ANOVA, *P* < 0.0001) (Fig. 5C). Therefore, the data suggest that the *L. longbeachae* T4SS is not solely responsible for inhibition of ROS generation during co-infections and that other inhibiting factors may be targeting alternative intracellular response pathways activated by *E. coli* instead of *L. pneumophila.* Altogether, while a lack of robust ROS production by neutrophils in response to *L. longbeachae* during solo-infection was independent of the T4SS, inhibition of ROS production during co-infection was largely dependent on the *L. longbeachae* T4SS.

### Failure of neutrophils to assemble the phagocytic NADPH oxidase complex in response to *L. longbeachae*

The catalytically active phagocyte NADPH oxidase complex is composed of membrane-bound and cytosolic subunits (52–56). Regulation of NADPH oxidase function in resting phagocytes is maintained by compartmentalizing the three membrane-bound subunits (gp91phox/NOX2, p22phox, and Rap1A) away from the cytosolic components (p40phox, p47phox, p67phox, and Rac2) of the NADPH oxidase complex (52–56). Upon activation of neutrophils, cytosolic components of the NADPH oxidase complex translocate to the membrane-bound subunits to form the catalytically active NADPH oxidase that catalyzes the conversion of oxygen to superoxide anions for generation of ROS (52–56). We determined whether *L. longbeachae* inhibition of neutrophil ROS generation was mediated by interfering with the assembly of the phagocytic NADPH oxidase complex.

**Fig 6:**
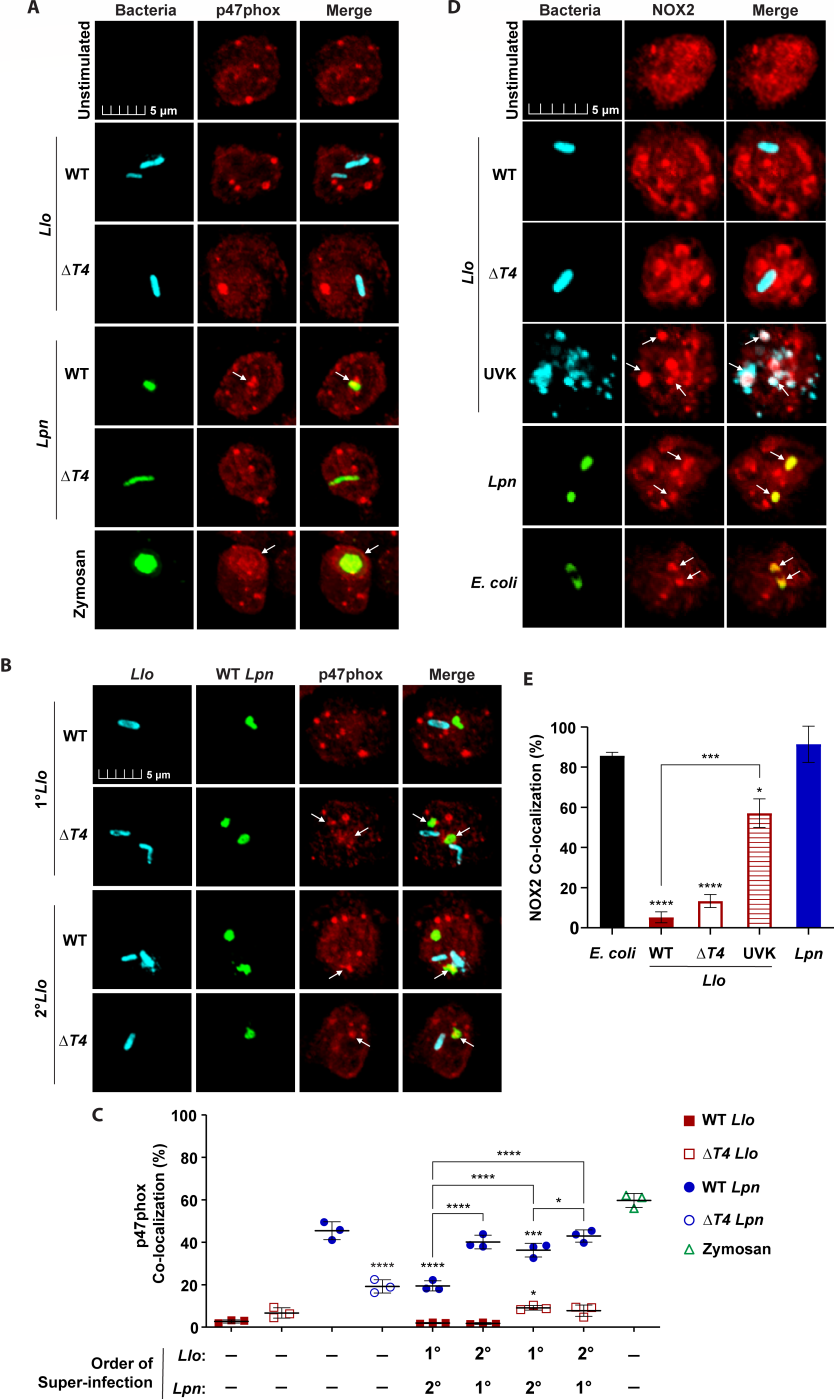
Failure of neutrophils to assemble the NADPH oxidase complex in response to *L. longbeachae*. To determine the assembly of the phagocyte NADPH oxidase complex at bacterial phagosomes, neutrophils were labeled with antibodies for cytosolic subunits and membrane-bound components of the NADPH oxidase complex. Neutrophils were solo-infected with WT or Δ*T4* bacterial strains for 15 min or subjected to 15 min primary infections (1°) followed by super-infection (2°) for an additional 15 minutes. Representative confocal images of cytosolic p47phox localization to phagosomes in neutrophils solo-infected (**A**) or super-infected (**B**). Bacteria were labeled with anti-*Llo* antibody (cyan) or anti-*Lpn* antibody (green). Zymosan A BioParticles Alexa Fluor 594 are shown in green. p47phox was labeled with anti-p47phox antibody (red). White arrows indicate positive co-localization of p47phox with pathogen-containing phagosomes. (**C**) Data are shown as mean percent co-localization of p47phox to *Llo* phagosomes (red) or *Lpn* phagosomes (blue) during infections. Zymosan was included as a positive control (green). ±SD, *n* = 3 (scatter plot dots). (**D**) Representative confocal images of the membrane-bound catalytic core gp91phox/NOX2 (NOX2) subunit co-localizing with phagosomal membranes in neutrophils solo-infected with WT or Δ*T4 Llo* strains, UV-killed (UVK) *Llo*, WT *Lpn*, or m-cherry *E. coli*. White arrows indicate positive co-localization of gp91phox/NOX2 with pathogen-containing phagosomes. Bacteria were labeled with anti-*Llo* antibody (cyan) or anti-*Lpn* antibody (green). The gp91phox/NOX2 was labeled with anti-gp91phox/NOX2 antibody (red). White arrows indicate positive co-localization of p47phox with pathogen-containing phagosomes. (**E**) Data are shown as mean percent co-localization of NOX2 to *Llo* phagosomes (red) or *Lpn* phagosomes (blue) during infections ± SD, *n* = 3. The data shown are representative of three independent biological repeats.

Neutrophils were assessed for recruitment of the NADPH oxidase cytosolic component p47phox to the pathogen-containing phagosomes during solo- and super-infections using confocal microscopy (Fig. 6). Neutrophils were stimulated with Zymosan A BioParticles as a positive control for assembly of the NADPH oxidase complex at the phagosome. Solo-infection of neutrophils for 15 min with WT and Δ*T4 L. longbeachae* resulted in less than 3% co-localization of p47phox cytosolic component to the WT and Δ*T4 Lo*-LCPs (Fig. 6A and C). By contrast, ~45% of the WT *Lp-*LCPs co-localized with p47phox, while Δ*T4 L. pneumophila* negative control phagosomes exhibited less than ~20% p47phox co-localization (ANOVA, *P* < 0.0001) (Fig. 6A and C). Therefore, the cytosolic components of the NADPH oxidase complex fail to be recruited to the *Lo*-LCP, and this is independent of the *L. longbeachae* T4SS.

To determine whether *L. longbeachae* exhibited *trans*-acting inhibition of p47phox recruitment to pathogen-containing phagosomes of co-inhabiting bacteria, we assessed p47phox co-localization during super-infections, as described above. Primary infection of neutrophils with WT *L. longbeachae* resulted in ~22% decreased co-localization of p47phox to WT *Lp-*LCPs (ANOVA, *P* < 0.0001), but the reduced co-localization of p47phox to the WT *Lp*-LCP was dependent on the *L. longbeachae* T4SS (ANOVA, *P* > 0.05) (Fig. 6B and C). While primary infection with WT *L. pneumophila* did not significantly increase p47phox co-localization to WT *Lo-*LCPs, co-localization of p47phox with Δ*T4 Lo-*LCPs mildly increased by ~6% (ANOVA, *P* < 0.0223) (Fig. 6A through C). Thus, the T4SS of *L. longbeachae* is required for protecting co-inhabiting bacteria from the recruitment of cytosolic components of the NADPH oxidase.

The membrane-bound NOX2/gp91phox catalytic core is responsible for the activity of the NADPH oxidase complex and is ubiquitously found in the plasma membrane, granule membranes, and membranes of endocytic compartments/phagosomes of phagocytic cells (57). Since the p47phox cytosolic subunit was excluded from *Lo*-LCPs, infected neutrophils were assessed for recruitment of the NOX2/gp91phox membrane-bound subunit to the pathogen-containing phagosomes. As UV-killed *L. longbeachae* did not inhibit ROS production by infected neutrophils, it was included to determine whether viability was necessary for inhibiting NOX2/gp91phox localization at the pathogen-containing phagosome. In addition, *E. coli* expressing an m-cherry plasmid or WT *L. pneumophila* were included as positive controls for the accumulation of NOX2/gp91phox at the pathogen-containing phagosome. Both the WT and Δ*T4 L. longbeachae* exhibited less than 10% NOX2/gp91phox co-localization to *Lo-*LCPs (Fig. 6D and E). By contrast, ~55% of phagosomes harboring UV-killed *L. longbeachae* co-localized with NOX2/gp91phox (ANOVA, *P* < 0.0007), while the *L. pneumophila* and *E. coli* positive controls exhibited 90% and 85% co-localization of NOX2/gp91phox to pathogen-containing phagosomes, respectively (Fig. 6D and E). Thus, similar to the exclusion of the cytosolic subunits of the phagocyte NADPH oxidase complex, the membrane-bound catalytic subunits are also excluded from the *Lo-*LCP independent of the *L. longbeachae* T4SS. This T4SS-independent exclusion of cytosolic and membrane-bound subunits of the NADPH oxidase complex from the *Lo*-LCP is consistent with the T4SS-independent failure in robust spatial ROS generation during *L. longbeachae* infection (Fig. 5). In addition, the T4SS of *L. longbeachae* is required for *trans*-acting exclusion of the cytosolic NADPH oxidase components from *Lp*-LCPs when co-inhabiting dual-infected cells. These results are also consistent with the T4SS-dependent inhibition of spatial ROS generation in response to *L. pneumophila* (Fig. 5A and B).

### Failure of robust degranulation and IL-8 production by neutrophils in response to *L. longbeachae*

Since *L. longbeachae* infection inhibited multiple intracellular microbicidal activities of neutrophils, we assessed whether *L. longbeachae* modulated the extracellular microbicidal response of neutrophils by measuring degranulation and pro-inflammatory cytokine production. Neutrophils were infected at an MOI of 5 with WT, Δ*T4,* and UV-killed strains of *L. longbeachae*. Aliquots of supernatants were collected at 30 min, 1 h, 4 h, 6 h, 12 h, and 20 h post*-*infection and prepared for ELISA analysis of specific granule release, azurophilic granule release, and IL-8 cytokine production. Kinetic data were further assessed by area under curve (AUC). Neutrophils were primed with 2 ng/mL of TNF-α and stimulated with 0.1 µM N-formyl-Me*t-*Leu-Phe (fMLP) as a control to induce degranulation. Cells were also infected with *E. coli* as a positive bacterial control for degranulation and IL-8 production.

It has been shown that neutrophils fail to degrade formalin-killed *L. pneumophila* (36). Since the extracellular pro-inflammatory response of human neutrophils to *L pneumophila* also has yet to be determined, the degradation experiments above (Fig. S7A through C) were repeated for formalin-killed, heat-killed, and UV-killed *L. pneumophila* to determine the best killed *L. pneumophila* control for induction of neutrophil response. Interestingly*,* UV-killed *L. pneumophila* exhibited 20% higher uptake compared to viable, formalin-killed, and heat-killed *L. pneumophila* (ANOVA, *P* < 0.0001) (Fig. S7D and E). Similar to UV-killed *L. longbeachae*, UV-killed *L. pneumophila* demonstrated ~75% degradation that was significantly higher than formalin-killed or heat-killed *L. pneumophila* strains (ANOVA, *P* < 0.0001) (Fig. S7D and F). Therefore, UV-killed *L. pneumophila* was included as a control to determine whether *L pneumophila* viability also influenced neutrophil degranulation or IL-8 production.

**Fig 7:**
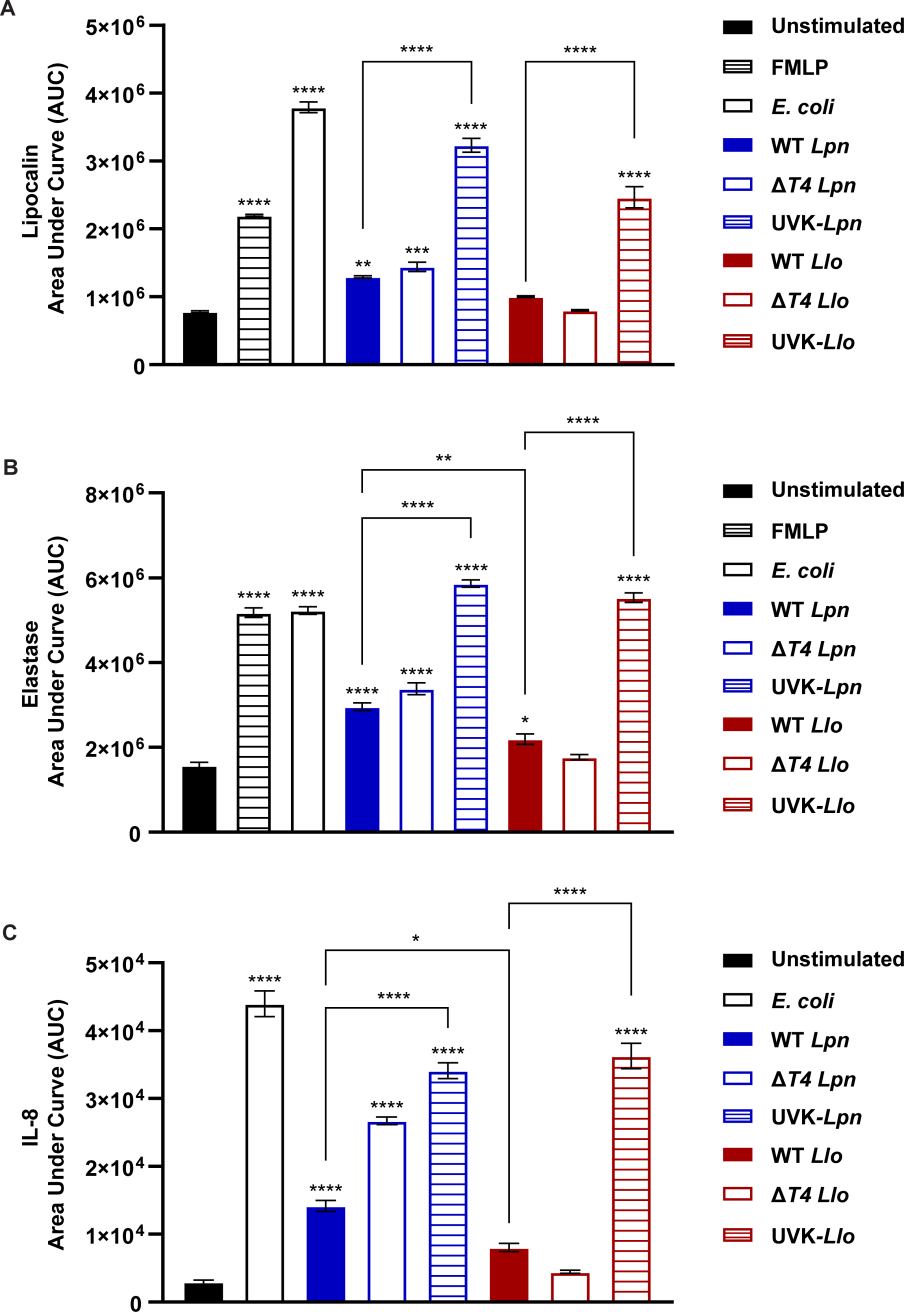
Failure of robust degranulation and IL-8 production by neutrophils in response to *L. longbeachae*. Neutrophils were infected with WT, Δ*T4*, or UV-killed (UVK) *Legionella* strains for up to 20 h at an MOI of 5. Cells were stimulated with fMLP or infected with *E. coli* at an MOI of 5 as additional positive controls. Aliquots of cell supernatants were collected at 0.5, 1, 4, 6, 12, and 20 h post-infection to quantify degranulation of specific granules using lipocalin as a marker (**A**), azurophilic granules using elastase as a marker (**B**), or IL-8 production (**C**) by ELISA. Data are shown as mean area under curve for kinetic degranulation and cytokine production during 20 h post-infection for unstimulated (black), fMLP-stimulated (black and white bars), *E. coli*-infected (black outline), *Lpn*-infected (blue), and *Llo*-infected (red) cells ± SEM, *n* = 3. The data shown are representative of three independent biological repeats.

To determine the degranulation of specific and azurophilic granules, we measured the concentration of lipocalin and elastase, respectively. Lipocalin release by neutrophils infected with WT and Δ*T4 L. longbeachae* were not significantly different from unstimulated cells (ANOVA, *P* > 0.05) (Fig. 7A; Fig. S8A, G, H). UV-killed *L. longbeachae* resulted in significantly higher concentrations of lipocalin than viable *L. longbeachae* (ANOVA, *P* < 0.0001), similar to the positive controls (Fig. 7A; Fig. S8A, B, C, G through I). Interestingly, infection with WT and Δ*T4 L. pneumophila* strains only induced a modest increase in lipocalin release compared to unstimulated cells (ANOVA, *P* < 0.0035, *P* < 0.0002) and UV-killed *L. pneumophila* resulted in significantly higher lipocalin release (ANOVA, *P* < 0.0001) (Fig. 7A; Fig. S8A through F), suggesting viable *L. pneumophila* may also dampen neutrophil degranulation. Similar to lipocalin, elastase release by neutrophils infected with Δ*T4 L. longbeachae* was not significantly different from unstimulated cells (ANOVA, *P* > 0.05), but there was a mildly significant increase in elastase release by cells infected with WT *L. longbeachae* (ANOVA, *P* < 0.0153) (Fig. 7B; Fig. S9A, G and H). However, the mild increase in elastase release observed for cells infected with WT *L. longbeachae* was not significantly different from cells infected with Δ*T4 L. longbeachae* (ANOVA, *P* > 0.05) (Fig. 7B; Fig. S9G and H). Neutrophils infected with UV-killed *L. longbeachae* demonstrated a significantly increased release of elastase compared to viable *L. longbeachae* (ANOVA, *P* < 0.0001) (Fig. 7B; Fig. 9G through I). In addition, infection of neutrophils with WT and Δ*T4 L. pneumophila* strains resulted in a significantly increased release of elastase compared to unstimulated cells (ANOVA, *P* < 0.0035, *P* < 0.0002), but was not to the level of the positive controls. On the other hand, infection of neutrophils UV-killed *L. pneumophila* resulted in significantly higher elastase release similar to the positive controls (ANOVA, *P* < 0.0001) (Fig. 7B; Fig. S9A through F). Altogether, the lack of robust degranulation by *L. longbeachae*-infected neutrophils is independent of the *L. longbeachae* T4SS (Fig. 7A and B; Fig. S8 and S9). Therefore, neutrophils fail to activate robust degranulation of specific and azurophilic granules in response to *L. longbeachae*, and this is independent of the *L. longbeachae* T4SS.

Activation of neutrophil pro-inflammatory signaling following infection was determined by measuring IL-8 concentration from collected supernatants. Infection with WT and Δ*T4 L. longbeachae* failed to induce significant IL-8 production from neutrophils compared to unstimulated cells (ANOVA, *P* > 0.05) (Fig. 7C; Fig. S10A, G, and H). Compared to viable *L. longbeachae,* UV-killed *L. longbeachae* and the *E. coli* positive control stimulated significant production of IL-8 from infected cells (ANOVA, *P* < 0.0001) (Fig. 7C; Fig. S10B and H). In addition, infection of neutrophils with WT, Δ*T4,* and UV-killed *L. pneumophila* strains resulted in IL-8 production compared to unstimulated cells (ANOVA, *P* < 0.0001) but IL-8 production of neutrophils induced by WT *L. pneumophila* was significantly less than the Δ*T4,* and UV-killed *L. pneumophila* strains (ANOVA, *P* < 0.0001) (Fig. 7C; Fig. S10A through E). Altogether, WT and Δ*T4 L. longbeachae* strains fail to induce robust IL-8 production by infected neutrophils. In addition, *L. pneumophila* may utilize its T4SS for dampening IL-8 production by infected cells. Overall, the data reveal that human neutrophils fail to activate a robust extracellular pro-inflammatory response to *L. longbeachae*, and this is independent of the *L. longbeachae* T4SS.

## DISCUSSION

**Fig 8:**
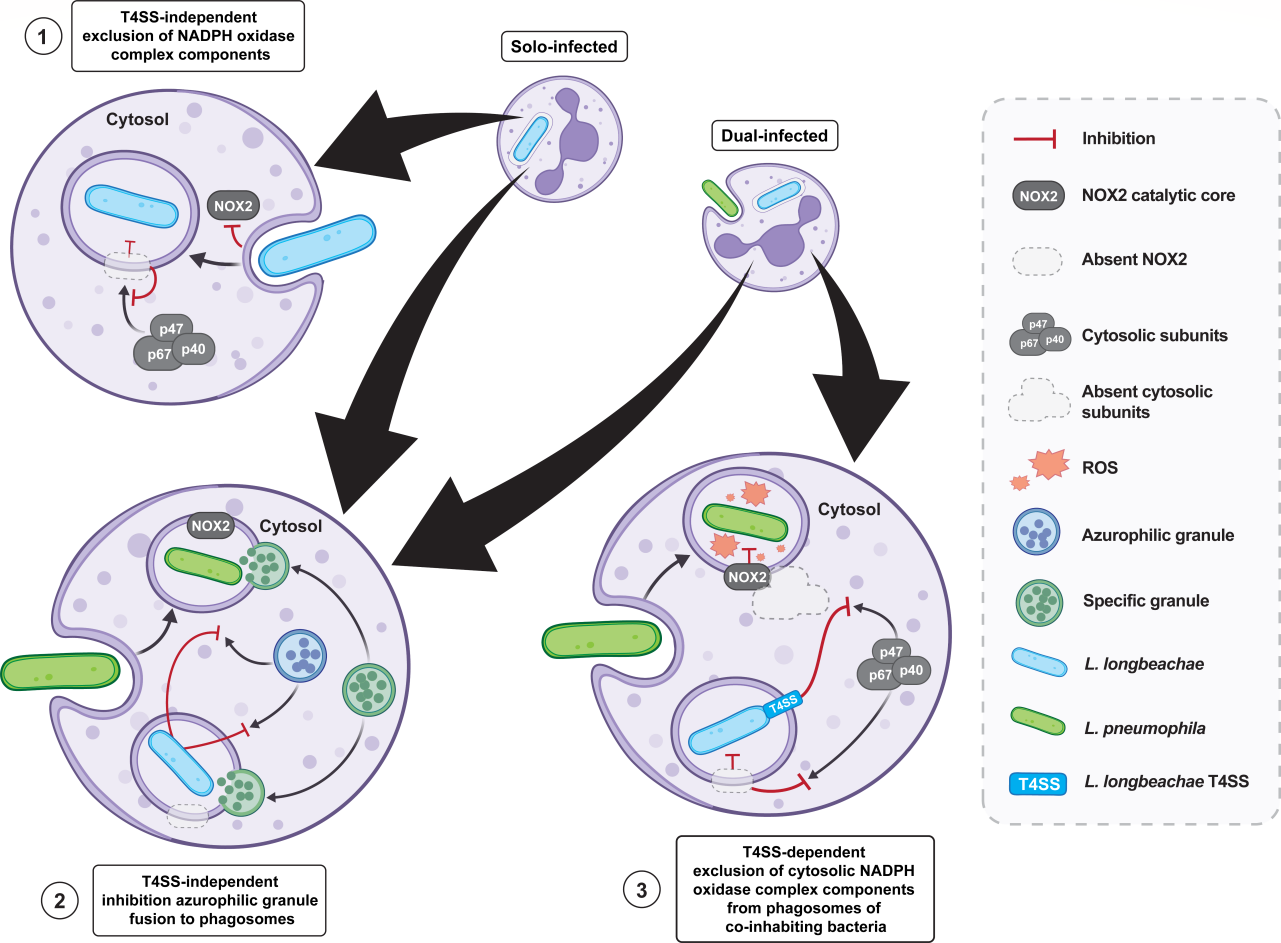
Three strategies employed by *L. longbeachae* for evasion and inhibition of microbicidal activities in neutrophils. Model of T4SS-independent and T4SS-dependent inhibition of neutrophil microbicidal activities by *L. longbeachae via* (1) T4SS-independent evasion of NADPH oxidase assembly at the *L. longbeachae* phagosome for inhibition of ROS production, (2) T4SS-independent inhibition of fusion of azurophilic granules to pathogen-containing phagosomes during solo-infection or dual-infections, and (3) T4SS-dependent inhibition of NADPH oxidase complex formation to intracellular phagosomes for impeding ROS generation during dual infections.

The mechanism of the T4SS-dependent rapid degradation of *L. pneumophila* by human neutrophils is mediated by the fusion of azurophilic granule to the pathogen-containing phagosome as well as the spatial assembly of the NADPH oxidase complex and ROS generation at the *L. pneumophila*-containing phagosome (36). Little is known regarding the response of human neutrophils to challenge by *L. longbeachae*. Our data show a clear divergence of *L. longbeachae*, which evades neutrophil restriction even when neutrophils are activated by other stimuli, indicating strong dominance of *L. longbeachae* in impeding the activation of neutrophils. Here, we reveal that *L. longbeachae* evasion of degradation by human neutrophils is mediated by the inhibition of major microbicidal processes. This pathogen evasion of degradation by neutrophils includes inhibiting the fusion of azurophilic granules to the pathogen-containing phagosomes, inhibiting the assembly of the NADPH oxidase complex and failing to generate ROS, and failing to induce robust pro-inflammatory responses. Remarkably, evasion of these microbicidal processes of neutrophils by *L. longbeachae* is T4SS independent. In addition, the T4SS was only required for *trans*-acting protection of co-inhabiting bacteria from ROS generation within the same neutrophil. Altogether, we propose a working model that *L. longbeachae* that employs three identified strategies for the evasion of various innate microbicidal processes of human neutrophils (see model in Fig. 8).

The generation of ROS by the catalytically active NADPH oxidase complex is one of the major mechanisms for pathogen degradation by neutrophils (52–56). Our data show that *L. longbeachae* utilizes a T4SS-independent mechanism for excluding membrane-bound subunits of the NADPH oxidase complex from its phagosome, resulting in failure to recruit cytosolic subunits of the NADPH oxidase complex and, thus, failure of neutrophils to generate robust ROS at the pathogen phagosome. However, in neutrophils co-inhabited by other bacteria, *L. longbeachae* demonstrates a *trans*-acting T4SS-dependent effect impeding recruitment of cytosolic subunits of the NADPH oxidase complex to phagosomes of co-inhabiting bacteria, leading to a T4SS-mediated inhibition of ROS generation. Based on this information, it is possible that the absence of cytosolic components of the NADPH oxidase complex from the *L. longbeachae* phagosome is a consequence of both T4SS-independent exclusion of the membrane-bound subunits as well as T4SS-dependent inhibition of pathways for translocation of cytosolic subunits throughout the cell cytosol (58). In addition, it is enigmatic that the *L. longbeachae* phagosome did not acquire the NOX2 catalytic core of the NADPH oxidase complex, as this component is present in membranes of specific granules that still fused to the *L. longbeachae* phagosome (42, 46, 51). Therefore, it is probable that the *L. longbeachae* phagosome may only acquire a subset of markers for specific granules by eliminating or selecting against them and, thus, disrupt the microbicidal processes directed toward the *L. longbeachae* phagosome.

As an environmental pathogen that has co-evolved with a diverse repertoire of environmental hosts and microbial competitors within the soil, it is unsurprising that *L. longbeachae* has acquired a variety of tools and strategies for modulating multiple host cell responses that are either T4SS dependent or T4SS independent (1, 24, 25). This is divergent from *L. pneumophila*, which triggers T4SS-dependent immune-metabolic reprogramming of neutrophils and engages their microbicidal mechanisms leading to rapid restriction of the pathogen (36). Remarkably, the exclusion of the membrane-bound subunits of the NADPH oxidase from the *L. longbeachae* phagosome is an exciting discovery previously reported for only two other pathogens that also interact with protist hosts in the soil environment, *Anaplasma phagocytophilum* and *Francisella tularensis* (59–64). In addition to repressing the accumulation of NOX2 from the phagosomal membrane, *A. phagocytophilum* also inhibits vacuolar acidification in a T4SS-dependent manner and dysregulates transcription of ROS-associated genes for inhibiting ROS generation (65). Interestingly, inhibition of neutrophil microbicidal activity by *F. tularensis* is similar to *L. longbeachae*, including inhibited fusion of azurophilic granules and suppression of ROS generation at the pathogen-containing phagosome (63, 66). It is possible that *A. phagocytophilum*, *F. tularensis*, and *L. longbeachae* share similar strategies for evading restriction by human neutrophils and promoting global interference of neutrophil microbicidal activities. On the other hand, it is more likely that idiosyncratic mechanisms are employed by these three pathogens to exclude the membrane-bound subunits of the NADPH oxidase from the pathogen-containing phagosome (67, 68). However, the potential factors involved in *A. phagocytophilum, F. tularensis,* and *L. longbeachae* exclusion of the membrane-bound subunits of the NADPH oxidase from the phagosomal membrane have yet to be discovered.

In addition to T4SS-independent inhibition of active NADPH oxidase complex assembly and ROS generation at its phagosome, *L. longbeachae* also exhibits a T4SS-independent strategy for impeding the fusion of azurophilic granules to its phagosome. Interestingly, the T4SS-independent strategy for inhibiting the fusion of azurophilic granules to pathogen-containing phagosomes is *trans*-acting throughout the cytosol of infected neutrophils. Thus, the divergent evolution of *L. longbeachae* through interactions with a different repertoire of environmental hosts has resulted in the pathogenic utilization of T4SS-independent strategies for inhibiting ROS generation and fusion of azurophilic granules to bacterial phagosomes.

It has been speculated that the enhanced severity of *L. longbeachae* infection and mortality in murine models is due to the lack of flagella or other pro-inflammatory-stimulating factors independent of the *L. longbeachae* T4SS, resulting in immune evasion and being termed “immunologically silent” (13). Since the T4SS of *L. longbeachae* is not required for inhibition of ROS generation or fusion of azurophilic granules at its phagosome, it is evident that *L. longbeachae* does not rely on T4SS effectors for evasion of degradation and suppression of neutrophil microbicidal activities. In addition, failure to activate a robust neutrophil pro-inflammatory response is also independent of the *L. longbeachae* T4SS, making it clear that virulence factors independent of the T4SS are responsible for *L. longbeachae* evasion and modulation of neutrophil microbicidal responses.

The T2SS of Gram-negative bacteria is known to deliver toxins, virulence factors, and hydrolytic enzymes to bacterial cell surfaces or extracellular spaces that enhance pathogenicity and modulate host cell responses (69–71). While the T2SS of *L. pneumophila* secretes up to 60 predicted proteins (24, 72, 73), the specific number of T2SS proteins for *L. longbeachae* is currently unknown. In addition, only 13 of the 60 predicted T2SS-secreted proteins for *L. pneumophila* are found in *L. longbeachae* (24, 72, 73). Since proteome analysis reveals the *L. longbeachae* genome encodes over 430 proteins harboring putative N-terminal signal peptides required for T2SS, it is possible that specific substrates of the *L. longbeachae* T2SS are involved in the evasion of degradation and/or inhibition of the neutrophil microbicidal activities. Thus, divergent evolution between *L. longbeachae* and *L. pneumophila* has resulted in these pathogens harboring significantly different T2SS and T4SS protein armories (24, 72).

Unlike *L. pneumophila, L. longbeachae* encodes a capsule that masks its LPS and likely contributes to the evasion of TLR-mediated phagocytosis and/or prevents stimulation of host immune cells (1, 12, 13, 74). The LPS of *L. pneumophila* is composed of complex fatty acids and is found to be a phase-variable virulence factor (75). While the LPS composition for *L. pneumophila and L. longbeachae* share similarities (76, 77), the role of the *L. longbeachae* LPS during infection has yet to be thoroughly explored. Thus, it is likely that the *L. longbeachae* capsule contributes to *L. longbeachae* evasion and inhibition of human neutrophil microbicidal activities. Interestingly, *L. longbeachae* is readily phagocytosed by human neutrophils with many bacteria per cell, suggesting that the *L. longbeachae* capsule may not be anti-phagocytic for these innate immune cells. In addition to this, it is also possible that neutrophils may phagocytose *L. longbeachae* through a non-inflammatory pathway, such as complement receptor 3 (CR3) or siglec pathways (78–81), that fails to engage cell activation or robust pro-inflammatory response seen with TLR stimulation. Based on the failure to induce robust degranulation and IL-8 production during *L. longbeachae* infection, we propose that *L. longbeachae* may passively enter neutrophils *via* a “silent” and non-inflammatory mode of entry, preventing significant activation of the host pro-inflammatory response prior to or in addition to the secretion of various virulence factors into the host cell cytosol (63, 82). Nevertheless, it is apparent that multiple T4SS-independent and T4SS-dependent strategies are working in synergy and are involved in the immune evasion of various microbicidal mechanisms by *L. longbeachae*.

Here, we provide novel and exciting evidence of three strategies employed by *L. longbeachae* for evading restriction by human neutrophils (see model Fig. 8): (i) T4SS-independent exclusion of membrane-bound components of the phagocyte NADPH oxidase complex on the *L. longbeachae*-containing phagosome resulting in the impeded assembly of the catalytically active NADPH oxidase complex and inhibited generation of ROS; (ii) T4SS-independent inhibition of fusion of azurophilic granules to both the *L. longbeachae* containing-phagosome and phagosomes of co-infecting bacteria; and (iii) T4SS-dependent *trans*-acting inhibition of assembly of the catalytically active NADPH oxidase complex resulting in inhibition of ROS generation. Thus, *L. longbeachae* is one of few environmental pathogens capable of evading degradation by human neutrophils and suppressing the microbicidal responses of these key innate immune cells for enhanced immune evasion, pathogenesis, and disease severity.

## MATERIALS AND METHODS

### Bacterial strains and cell lines

WT and Δ*dotB* (Δ*T4*) *L. longbeachae* strain NSW150 were a generous gift from Dr. Stephanie Shames of Kansas State University. *L. pneumophila* strain AA100/130b (ATCC BAA-74), the Δ*dotA* (Δ*T4*) mutant of *L. pneumophila*, *L. longbeachae* strain NSW150, the Δ*T4* mutant of *L. longbeachae* NSW150, and *L. longbeachae* strain D4968 were grown on BCYE agar for 3 days. K12 *E. coli* strain expressing m-cherry plasmid was used for selected experiments. Before infections, bacteria were grown overnight in BYE to the post-exponential phase as described previously (36).

Human neutrophils were isolated from the venous blood of healthy donors using Dextran sedimentation and plasma-Percoll gradients as described previously (83). Flow cytometry evaluation showed ≥96% of recovered cells were neutrophils. Trypan blue exclusion indicated cell viability of >97%. Superoxide assay confirmed a lack of premature cell activation following the isolation with plasma-Percoll gradient method. Donor recruitment and blood draws were in accordance with the guidelines approved by the institutional review board of the University of Louisville (IRB#04.0358).

### Preparation of acid-washed coverslips

12 mm coverslips (Fisher, 12-545-80) were heated in a covered beaker containing 1 N HCL at 60°C for 4 h. Coverslips were washed several times with ddH_2_O followed by three washes with 70% ethanol. After the last wash of 70% ethanol, coverslips were washed twice more with 95% ethanol. The remaining ethanol was aspirated, and coverslips were dried prior to storage or use.

### Opsonization of bacterial strains for neutrophil infection

For infection of neutrophils, bacterial strains were opsonized with human serum following the protocol described previously (36). A total of 1 × 10^8^ bacteria were added to 500 µL of Hanks’ balanced salt solution (HBSS) containing 10% pooled human serum and incubated for 30 min at room temperature. Following incubation, opsonized bacteria were pelleted by centrifugation and resuspended in sterile water.

### Bacterial uptake and survival in neutrophils

To observe the uptake, degradation, or maintained morphology of bacterial strains during neutrophil infections, confocal microscopy was used. Neutrophils were plated into 24-well plates containing acid-washed glass coverslips (3 × 10^6^ cells per well) and infected with post-exponential phase WT *L. pneumophila*, ΔT4 *L. pneumophila*, WT *L. longbeachae,* or ΔT4 *L. longbeachae* at an MOI of 1 for experiments as described below. Phagocytosis was synchronized at 600× *g* and 14°C for 4 min, and cells were incubated at 37°C in 5% CO_2_.

For assessing uptake and degradation during solo-infections, neutrophils were infected for 15 min with opsonized or un-opsonized bacteria. At 15 min post-infection, cells were fixed with 4% paraformaldehyde. Extracellular bacteria were labeled with goat anti-*L. pneumophila* antibody (1:1,000 dilution) or rabbit anti-*L. longbeachae* antibody. Cells were permeabilized with 0.1% Triton-100 as previously described (36). Intracellular bacteria were labeled with rabbit anti-*L. pneumophila* antibody (1:1,000 dilution) or chicken anti-*L. longbeachae* antibody (1:4,000 dilution). After primary labeling, cells were labeled with donkey anti-rabbit Alexa Fluor 488 (1:2,000 dilution, Invitrogen #A32790), donkey anti-chicken Alexa Fluor 555 (1:2,000 dilution, Invitrogen #A78949), donkey anti-goat Alexa Fluor 555 (1:2,000, Invitrogen # A21432), and DAPI (1:5,000 dilution, Millipore Sigma #80051-386). Cells containing intracellular bacteria labeled with only donkey anti-rabbit Alexa Fluor 488 (*L. pneumophila*) or donkey anti-chicken Alexa Fluor 555 (*L. longbeachae*) were quantified from total cells in the field of view. The morphology of intracellular bacteria was analyzed by confocal microscopy.

During all other solo-infections or co-infections, cells were fixed at 15 min post-infection with 4% paraformaldehyde and permeabilized with 0.1% Triton-100 as previously described (36). Cells were labeled with rabbit anti-*L. pneumophila* antibody (1:1,000 dilution) and chicken anti-*L. longbeachae* antibody (1:4,000 dilution). After primary labeling, cells were labeled with donkey anti-rabbit Alexa Fluor 488 (1:2,000 dilution, Invitrogen #A32790), donkey anti-chicken Alexa Fluor 555 (1:2,000 dilution, Invitrogen #A78949), and DAPI (1:5,000 dilution, Millipore Sigma #80051-386). Intracellular bacterial morphology was analyzed by confocal microscopy. To determine whether bacterial morphology correlated with the killing or survival of intracellular bacteria, recoverable CFUs were determined as previously described at 5, 15, 60, and 120 min post-infection (36).

For CFU analysis of co-infections, chloramphenicol-resistant WT *L. pneumophila* and kanamycin-resistant *L. longbeachae* strains were grown on respective antibiotic BCYE plates for 3 days. For specified experiments, bacterial strains were grown in BYE broth with respective antibiotics overnight prior to opsonization and infection. Recoverable CFUs from solo- and co-infected were determined at 5, 15, 60, and 120 min post-infection by plating total cell lysates (described above) on chloramphenicol or kanamycin BCYE square plates.

### Confocal microscopy of neutrophils

Following infections, neutrophils were subjected to confocal scanning and imaging with Olympus FV3000. Images to determine bacterial uptake, bacterial morphology, and co-localization of markers with bacterial phagosomes in neutrophil cytosol were taken as Z-stacks in 0.25 µm slices. To differentiate between degraded and rod-shaped bacteria on a vertical or Z-plane, Z-stacks were examined with volume field. Bacterial phagosomes positive or negative for cell markers were quantified using ratio intensity in LUT. Bacterial uptake and degradation during super-infections were determined by quantifying the number solo- and dual-infected cells relative to total cells and internal bacterial morphology within each super-infection group, respectively.

### Preparation of samples for TEM

Neutrophils were infected for 1 h with bacterial strains. Pelleted samples were placed in 2% glutaraldehyde, 0.1 M sodium cacodylate buffer with a pH of 7.4 fixative for 24 h at 4°C. Samples were stained with 1% osmium tetroxide for 1 h followed by ethanol dehydration steps of 30%, 50%, 70%, 90%, and anhydrous ethanol three times. Embedding was performed with Spurr’s resin and anhydrous ethanol at 1:1 and 3:1 for 1 h followed by curing at 70°C overnight in 100% resin. Embedded samples were sectioned down to 80 nm with a Leica UC-7 Ultramicrotome and placed onto nickel slot grids with formvar supporting film. Grids were post-stained with 2% uranyl acetate and 3% lead citrate (Leica Ultrostain II) and imaged with a Hitachi HT-7700 TEM at 80kV.

### Super-infection of neutrophils

Neutrophils were adhered to acid-washed coverslips for 30 min and then subjected to primary infection with opsonized bacteria at an MOI of 1. Phagocytosis was synchronized at 600 × *g* and 14°C for 4 min, and cells were incubated at 37°C in 5% CO_2_ for 15 min. For secondary infection and solo-infection controls, opsonized bacteria were added to desired wells at an MOI of 1. Following the addition of bacteria, phagocytosis was synchronized, and cells were incubated at 37°C in 5% CO_2_ for 15 min. At 15 min post-infection, cells were fixed and permeabilized with 0.1% Triton-100 as previously described (36). Cells were labeled with rabbit anti-*L. pneumophila* antibody (1:1,000 dilution) and chicken anti-*L. longbeachae* antibody (1:4,000 dilution). After primary labeling, cells were labeled with donkey anti-rabbit Alexa Fluor 488 (1:2,000 dilution, Invitrogen #A32790), donkey anti-chicken Alexa Fluor 555 (1:2,000 dilution, Invitrogen #A78949), and DAPI (1:5,000 dilution, Millipore Sigma #80051-386). Intracellular bacterial morphology was analyzed by confocal microscopy. Assessment and antibodies used for granule recruitment and NADPH oxidase activity following super-infections are described below.

### Granule fusion and recruitment of NADPH oxidase components to bacterial phagosomes

Recruitment of specific granules, azurophilic granules, and the NADPH oxidase complex components to the bacterial phagosomes during superinfection was determined by infecting neutrophils and following the super-infection procedure above. For assessment of Gp91phox/NOX2, m-cherry *E. coli* was included at an MOI of 1 as a positive control. In addition, Zymosan A BioParticles Alexa Fluor 594 (2 µg/µL, Invitrogen #Z23374) was used as a positive control for granule and p47phox marker recruitment.

After fixation and permeabilization, cells to be analyzed for granule fusion were labeled with goat anti-*L. pneumophila* antibody (1:1,000 dilution), chicken anti-*L. longbeachae* antibody (1:4,000 dilution), rabbit anti-lactoferrin antibody (1:100 dilution, Abcam #Ab109216), rabbit anti-NGAL antibody (1:100 dilution, ThermoFisher #PA5-79589), rabbit anti-CEACAM8/Cd66b antibody (1:200 dilution, ThermoFisher # PA5-104296), and mouse anti-elastase antibody (1:100 dilution, Abcam #Ab254178). After primary labeling, cells were labeled with donkey anti-rabbit Alexa Fluor 405 (1:250 dilution, Invitrogen #A48257), donkey anti-mouse Alexa Fluor 488 (1:2,000 dilution, Invitrogen #A32766), donkey anti-goat Alexa Fluor 555 (1:2,000 dilution, Invitrogen #A21432), and donkey anti-chicken Alexa Fluor 647 (1:2,000 dilution, Invitrogen #A78952).

For the analysis of p47phox recruitment, cells were labeled using chicken anti-*L. longbeachae* antibody (1:4,000 dilution), rabbit anti-*L. pneumophila* antibody (1:1,000 dilution), and goat anti-p47phox antibody (1:100 dilution, Abcam #Ab166930). Gp91phox/NOX2 localization was determined by labeling cells with rabbit anti-NOX2 antibody (1:200 dilution, ThermoFisher #PA5-141135) in addition to chicken anti-*L. longbeachae* antibody (1:4,000 dilution) and goat anti-*L. pneumophila* antibody (1:1,000 dilution). After primary labeling, cells were labeled with DAPI (1:5,000 dilution, Millipore Sigma #80051-386), donkey anti-chicken Alexa Fluor 488 (1:2,000 dilution, Invitrogen #AA78948), donkey anti-goat Alexa Fluor Plus 555 (1:1,000 dilution, Invitrogen #A32773), and donkey anti-rabbit Alexa Fluor 647 (1:2,000 dilution, Invitrogen #A31573). Labeled cells were analyzed by confocal microscopy for co-localization of lactoferrin, elastase, p47phox, or NOX2 with bacterial phagosomes. Quantifications were performed by counting bacterial phagosomes showing positive co-localization out of total bacteria.

### Respiratory burst of neutrophils

Kinetics of ROS production *via* NADPH oxidase activity was measured using chemiluminescence with 125 µM luminol in the presence of 75 µg/mL superoxide dismutase and 20 µg/mL horseradish peroxidase (HRP) (84, 85). 4 × 10^5^ neutrophils were plated in 96-well white plates. Zymosan A BioParticles Alexa Fluor 594 (2 µg/µL, Invitrogen #Z23374) was used as a positive control for neutrophil ROS production (data not included) (86). Neutrophils were solo- or co-infected with WT or ΔT4 *L. longbeachae*, UV-killed *L. longbeachae*, WT *L. pneumophila*, or *E. coli* at an MOI of 5 each or an additional MOI of 10 for WT *L. pneumophila* as specified in text. After the addition of stimuli, neutrophils were spun at room temperature at 600 × *g* for 4 min and immediately placed in a Synergy H1 microplate reader (BioTek, Winooski, VT, USA). Total integrated relative light units (RLUs) at 37°C were determined for 80 min using SoftMax Pro software (Molecular Devices). The data are represented as area under curve from three different experiments.

### Degranulation and cytokine analysis of neutrophils

Ultra-pure neutrophils were primed with 2 ng/ml TNF-α and stimulated with 0.1 µM N-formyl-Met-Leu-Phe (fMLP) as a positive control or infected with WT, ΔT4, or UV-killed *L. longbeachae*, WT *L. pneumophila*, ΔT4 *L. pneumophila*, or *E. coli* at 37°C for 0.5, 1, 4, 6, 12, and 20 h in 12-well plates. At each timepoint, supernatants were collected and filtered through 0.2 µm Acrodisc syringe filters (Pall Corporation). Prior to storage at −80°C, samples were treated with Halt protease phosphatase inhibitor (#78440, ThermoFisher). Assessment of specific granule and azurophilic granule release was determined by ELISA using the Human Lipocalin-2/NGAL DuoSet kit (#DY1757, R&D Systems) and the Human Neutrophil Elastase/ELA2 DuoSet kit (#DY9167, R&D systems), respectively, in accordance with the manufacturer’s instructions. Assessment of IL-8 production in cell supernatants was determined by ELISA using Human IL-8/CXCL8 DuoSet ELISA kit (#DY208, R&D Systems) in accordance with the manufacturer’s instructions.

### Ultra-purification of neutrophils

To ensure 99.99% cell purity for cytokine analysis, neutrophils isolated from plasma-Percoll gradients were resuspended in RoboSep Buffer (#20104, STEMCELL Technologies), and transferred into 5 mL Falcon polystyrene round-bottom tubes. EasySep Human Neutrophil Isolation Cocktail from EasySep Human Neutrophil Isolation Kit (#17957, STEMCELL Technologies) was added to the cell solution and incubated for 10 min. Following the cocktail, EasySep Dextran RapidSpheres were added to cell suspension and incubated for 20 min. Additional RoboSep Buffer was added to the cell suspension and mixed for a final volume of 2.5 mL. Cell suspension tubes were placed into an EasySep Magnet (#18000, STEMCELL Technologies) for 10 min before carefully removing cell suspension and checking neutrophil viability and purity.

### Statistical analysis

Data presented as mean and SD of three experimental replicates (*n* = 3). Differences between groups were compared by means of Student’s *t*-test or multiple comparisons with ANOVA as specified in the text. Statistical significances are reported in the text, figures, and figure legends. Statistical analysis was performed in GraphPad PRISM 10.

## SUPPLEMENTAL MATERIAL

Supplemental material mbio.03274-24-s0001.pdf Fig. S1-S10 and Table S1.

ASM does not own the copyrights to Supplemental Material that may be linked to, or accessed through, an article. The authors have granted ASM a non-exclusive, world-wide license to publish the Supplemental Material files. Please contact the corresponding author directly for reuse.
