# Supplementary material for: Inhibition and evasion of neutrophil microbicidal responses by Legionella longbeachae
Source: mBio. 2024 Dec 16;16(2):e03274-24. doi: 10.1128/mbio.03274-24 (PMC11796426; doi:10.1128/mbio.03274-24)
Supplement: Supplemental material — Fig. S1-S10 and Table S1. [file mbio.03274-24-s0001.pdf]

Supplemental Table 1: Multiple comparisons for Figure S5

Compare cell means with others in its row and its column

Number of families	12
Number of comparisons per row family	21
Number of comparisons per column family	10
Alpha	0.05

Tukey's multiple comparisons test	Mean Diff.	95.00% CI of diff.	Below thres	Summary	Adjusted P Value
Inoculum					
WT Llo vs. ΔT4SS Llo	0.09436	-0.1981 to 0.3868	No	ns	0.9568
WT Llo vs. WT Llo + WT Lpn	0.05532	-0.2372 to 0.3478	No	ns	0.9973
WT Llo vs. ΔT4SS Llo + WT Lpn	-0.04261	-0.3351 to 0.2499	No	ns	0.9994
WT Llo vs. WT Lpn	0.07624	-0.2162 to 0.3687	No	ns	0.9851
WT Llo vs. WT Llo + WT Lpn	0.08763	-0.2049 to 0.3801	No	ns	0.9699
WT Llo vs. ΔT4SS Llo + WT Lpn	0.1381	-0.1544 to 0.4306	No	ns	0.7818
ΔT4SS Llo vs. WT Llo + WT Lpn	-0.03904	-0.3315 to 0.2534	No	ns	0.9996
ΔT4SS Llo vs. ΔT4SS Llo + WT Lpn	-0.137	-0.4295 to 0.1555	No	ns	0.7884
ΔT4SS Llo vs. WT Lpn	-0.01812	-0.3106 to 0.2744	No	ns	>0.9999
ΔT4SS Llo vs. WT Llo + WT Lpn	-0.00673	-0.2992 to 0.2857	No	ns	>0.9999
ΔT4SS Llo vs. ΔT4SS Llo + WT Lpn	0.04376	-0.2487 to 0.3362	No	ns	0.9993
WT Llo + WT Lpn vs. ΔT4SS Llo + WT Lpn	-0.09793	-0.3904 to 0.1946	No	ns	0.9486
WT Llo + WT Lpn vs. WT Lpn	0.02092	-0.2716 to 0.3134	No	ns	>0.9999
WT Llo + WT Lpn vs. WT Llo + WT Lpn	0.0323	-0.2602 to 0.3248	No	ns	0.9999
WT Llo + WT Lpn vs. ΔT4SS Llo + WT Lpn	0.0828	-0.2097 to 0.3753	No	ns	0.9773
ΔT4SS Llo + WT Lpn vs. WT Lpn	0.1189	-0.1736 to 0.4113	No	ns	0.8786
ΔT4SS Llo + WT Lpn vs. WT Llo + WT Lpn	0.1302	-0.1622 to 0.4227	No	ns	0.8249
ΔT4SS Llo + WT Lpn vs. ΔT4SS Llo + WT Lpn	0.1807	-0.1118 to 0.4732	No	ns	0.5029
WT Lpn vs. WT Llo + WT Lpn	0.01138	-0.2811 to 0.3039	No	ns	>0.9999
WT Lpn vs. ΔT4SS Llo + WT Lpn	0.06188	-0.2306 to 0.3544	No	ns	0.9951
WT Llo + WT Lpn vs. ΔT4SS Llo + WT Lpn	0.05049	-0.2420 to 0.3430	No	ns	0.9984

15 min

WT Llo vs. WT Lpn	-0.2252	-0.5177 to 0.06724	No	ns	0.2412
WT Llo vs. WT Llo + WT Lpn	0.1309	-0.1616 to 0.4234	No	ns	0.8212
WT Llo vs. ΔT4SS Llo + WT Lpn	0.03491	-0.2576 to 0.3274	No	ns	0.9998
ΔT4SS Llo vs. WT Llo + WT Lpn	0.06461	-0.2279 to 0.3571	No	ns	0.9938
ΔT4SS Llo vs. ΔT4SS Llo + WT Lpn	-0.01114	-0.3036 to 0.2813	No	ns	>0.9999
ΔT4SS Llo vs. WT Lpn	-0.2081	-0.5006 to 0.08436	No	ns	0.3303
ΔT4SS Llo vs. WT Llo + WT Lpn	0.1481	-0.1444 to 0.4405	No	ns	0.7219
ΔT4SS Llo vs. ΔT4SS Llo + WT Lpn	0.05203	-0.2405 to 0.3445	No	ns	0.9981
WT Llo + WT Lpn vs. ΔT4SS Llo + WT Lpn	-0.07575	-0.3682 to 0.2167	No	ns	0.9856
WT Llo + WT Lpn vs. WT Lpn	-0.2727	-0.5652 to 0.01976	No	ns	0.0836
WT Llo + WT Lpn vs. WT Llo + WT Lpn	0.08345	-0.2090 to 0.3759	No	ns	0.9764
WT Llo + WT Lpn vs. ΔT4SS Llo + WT Lpn	-0.01257	-0.3051 to 0.2799	No	ns	>0.9999
ΔT4SS Llo + WT Lpn vs. WT Lpn	-0.197	-0.4895 to 0.09551	No	ns	0.3969
ΔT4SS Llo + WT Lpn vs. WT Llo + WT Lpn	0.1592	-0.1333 to 0.4517	No	ns	0.6493
ΔT4SS Llo + WT Lpn vs. ΔT4SS Llo + WT Lpn	0.06317	-0.2293 to 0.3557	No	ns	0.9945
WT Lpn vs. WT Llo + WT Lpn	0.3562	0.06369 to 0.6487	Yes	**	0.0075
WT Lpn vs. ΔT4SS Llo + WT Lpn	0.2602	-0.03233 to 0.5526	No	ns	0.1135
WT Llo + WT Lpn vs. ΔT4SS Llo + WT Lpn	-0.09602	-0.3885 to 0.1965	No	ns	0.9531
1 hr					
WT Llo vs. ΔT4SS Llo	0.0323	-0.2602 to 0.3248	No	ns	0.9999
WT Llo vs. WT Llo + WT Lpn	0.02232	-0.2702 to 0.3148	No	ns	>0.9999
WT Llo vs. ΔT4SS Llo + WT Lpn	-0.1274	-0.4199 to 0.1651	No	ns	0.8393
WT Llo vs. WT Lpn	0.1197	-0.1728 to 0.4122	No	ns	0.8751
WT Llo vs. WT Llo + WT Lpn	0.01525	-0.2772 to 0.3077	No	ns	>0.9999
WT Llo vs. ΔT4SS Llo + WT Lpn	0.1227	-0.1698 to 0.4151	No	ns	0.8618
ΔT4SS Llo vs. WT Llo + WT Lpn	-0.00999	-0.3025 to 0.2825	No	ns	>0.9999
ΔT4SS Llo vs. ΔT4SS Llo + WT Lpn	-0.1597	-0.4522 to 0.1328	No	ns	0.6461
ΔT4SS Llo vs. WT Lpn	0.08737	-0.2051 to 0.3799	No	ns	0.9703
ΔT4SS Llo vs. WT Llo + WT Lpn	-0.01705	-0.3095 to 0.2754	No	ns	>0.9999
ΔT4SS Llo vs. ΔT4SS Llo + WT Lpn	0.09036	-0.2021 to 0.3828	No	ns	0.965
WT Llo + WT Lpn vs. ΔT4SS Llo + WT Lpn	-0.1497	-0.4422 to 0.1428	No	ns	0.7115
WT Llo + WT Lpn vs. WT Lpn	0.09736	-0.1951 to 0.3898	No	ns	0.95
WT Llo + WT Lpn vs. WT Llo + WT Lpn	-0.00706	-0.2995 to 0.2854	No	ns	>0.9999
WT Llo + WT Lpn vs. ΔT4SS Llo + WT Lpn	0.1003	-0.1921 to 0.3928	No	ns	0.9424
ΔT4SS Llo + WT Lpn vs. WT Lpn	0.2471	-0.04543 to 0.5395	No	ns	0.1531
ΔT4SS Llo + WT Lpn vs. WT Llo + WT Lpn	0.1426	-0.1498 to 0.4351	No	ns	0.7553

ΔT4SS Llo + WT Lpn vs. ΔT4SS Llo + WT Lpn	0.25	-0.04244 to 0.5425	No	ns	0.1433
WT Lpn vs. WT Llo + WT Lpn	-0.1044	-0.3969 to 0.1881	No	ns	0.9308
WT Lpn vs. ΔT4SS Llo + WT Lpn	0.002985	-0.2895 to 0.2955	No	ns	>0.9999
WT Llo + WT Lpn vs. ΔT4SS Llo + WT Lpn	0.1074	-0.1851 to 0.3999	No	ns	0.9215
2 hr					
WT Llo vs. ΔT4SS Llo	-0.00408	-0.2966 to 0.2884	No	ns	>0.9999
WT Llo vs. WT Llo + WT Lpn	-0.06543	-0.3579 to 0.2271	No	ns	0.9934
WT Llo vs. ΔT4SS Llo + WT Lpn	-0.1408	-0.4333 to 0.1517	No	ns	0.7662
WT Llo vs. WT Lpn	0.3626	0.07016 to 0.6551	Yes	**	0.0061
WT Llo vs. WT Llo + WT Lpn	0.3101	0.01760 to 0.6026	Yes	*	0.0306
WT Llo vs. ΔT4SS Llo + WT Lpn	0.4916	0.1991 to 0.7841	Yes	****	<0.0001
ΔT4SS Llo vs. WT Llo + WT Lpn	-0.06135	-0.3538 to 0.2311	No	ns	0.9953
ΔT4SS Llo vs. ΔT4SS Llo + WT Lpn	-0.1367	-0.4292 to 0.1558	No	ns	0.7898
ΔT4SS Llo vs. WT Lpn	0.3667	0.07423 to 0.6592	Yes	**	0.0053
ΔT4SS Llo vs. WT Llo + WT Lpn	0.3142	0.02168 to 0.6066	Yes	*	0.0272
ΔT4SS Llo vs. ΔT4SS Llo + WT Lpn	0.4957	0.2032 to 0.7882	Yes	****	<0.0001
WT Llo + WT Lpn vs. ΔT4SS Llo + WT Lpn	-0.07537	-0.3679 to 0.2171	No	ns	0.986
WT Llo + WT Lpn vs. WT Lpn	0.4281	0.1356 to 0.7206	Yes	***	0.0006
WT Llo + WT Lpn vs. WT Llo + WT Lpn	0.3755	0.08303 to 0.6680	Yes	**	0.004
WT Llo + WT Lpn vs. ΔT4SS Llo + WT Lpn	0.557	0.2645 to 0.8495	Yes	****	<0.0001
ΔT4SS Llo + WT Lpn vs. WT Lpn	0.5034	0.2110 to 0.7959	Yes	****	<0.0001
ΔT4SS Llo + WT Lpn vs. WT Llo + WT Lpn	0.4509	0.1584 to 0.7434	Yes	***	0.0003
ΔT4SS Llo + WT Lpn vs. ΔT4SS Llo + WT Lpn	0.6324	0.3399 to 0.9249	Yes	****	<0.0001
WT Lpn vs. WT Llo + WT Lpn	-0.05255	-0.3450 to 0.2399	No	ns	0.998
WT Lpn vs. ΔT4SS Llo + WT Lpn	0.129	-0.1635 to 0.4214	No	ns	0.8315
WT Llo + WT Lpn vs. ΔT4SS Llo + WT Lpn	0.1815	-0.1110 to 0.4740	No	ns	0.4977
20 hr					
WT Llo vs. ΔT4SS Llo	-0.159	-0.4515 to 0.1334	No	ns	0.6504
WT Llo vs. WT Llo + WT Lpn	-0.04165	-0.3341 to 0.2508	No	ns	0.9995
WT Llo vs. ΔT4SS Llo + WT Lpn	-0.1197	-0.4122 to 0.1728	No	ns	0.8751
WT Llo vs. WT Lpn	1.391	1.099 to 1.684	Yes	****	<0.0001
WT Llo vs. WT Llo + WT Lpn	1.333	1.040 to 1.625	Yes	****	<0.0001
WT Llo vs. ΔT4SS Llo + WT Lpn	1.384	1.092 to 1.677	Yes	****	<0.0001
ΔT4SS Llo vs. WT Llo + WT Lpn	0.1174	-0.1751 to 0.4099	No	ns	0.8847
ΔT4SS Llo vs. ΔT4SS Llo + WT Lpn	0.03937	-0.2531 to 0.3319	No	ns	0.9996

ΔT4SS Llo vs. WT Lpn	1.55	1.258 to 1.843	Yes	****	<0.0001
ΔT4SS Llo vs. WT Llo + WT Lpn	1.492	1.199 to 1.784	Yes	****	<0.0001
ΔT4SS Llo vs. ΔT4SS Llo + WT Lpn	1.543	1.251 to 1.836	Yes	****	<0.0001
WT Llo + WT Lpn vs. ΔT4SS Llo + WT Lpn	-0.07803	-0.3705 to 0.2145	No	ns	0.9832
WT Llo + WT Lpn vs. WT Lpn	1.433	1.140 to 1.725	Yes	****	<0.0001
WT Llo + WT Lpn vs. WT Llo + WT Lpn	1.374	1.082 to 1.667	Yes	****	<0.0001
WT Llo + WT Lpn vs. ΔT4SS Llo + WT Lpn	1.426	1.133 to 1.718	Yes	****	<0.0001
ΔT4SS Llo + WT Lpn vs. WT Lpn	1.511	1.218 to 1.803	Yes	****	<0.0001
ΔT4SS Llo + WT Lpn vs. WT Llo + WT Lpn	1.452	1.160 to 1.745	Yes	****	<0.0001
ΔT4SS Llo + WT Lpn vs. ΔT4SS Llo + WT Lpn	1.504	1.211 to 1.796	Yes	****	<0.0001
WT Lpn vs. WT Llo + WT Lpn	-0.05825	-0.3507 to 0.2342	No	ns	0.9965
WT Lpn vs. ΔT4SS Llo + WT Lpn	-0.00695	-0.2994 to 0.2855	No	ns	>0.9999
WT Llo + WT Lpn vs. ΔT4SS Llo + WT Lpn	0.0513	-0.2412 to 0.3438	No	ns	0.9983
WT Llo					
Inoculum vs. 15 min	0.3015	0.03170 to 0.5713	Yes	*	0.0209
Inoculum vs. 1 hr	0.5066	0.2368 to 0.7764	Yes	****	<0.0001
Inoculum vs. 2 hr	0.6263	0.3565 to 0.8961	Yes	****	<0.0001
Inoculum vs. 20 hr	0.5635	0.2937 to 0.8333	Yes	****	<0.0001
15 min vs. 1 hr	0.2051	-0.06465 to 0.4749	No	ns	0.2195
15 min vs. 2 hr	0.3248	0.05503 to 0.5946	Yes	*	0.0104
15 min vs. 20 hr	0.262	-0.007748 to 0.5318	No	ns	0.0611
1 hr vs. 2 hr	0.1197	-0.1501 to 0.3895	No	ns	0.7269
1 hr vs. 20 hr	0.0569	-0.2129 to 0.3267	No	ns	0.9761
2 hr vs. 20 hr	-0.06278	-0.3326 to 0.2070	No	ns	0.9658
ΔT4SS Llo					
Inoculum vs. 15 min	0.19	-0.07979 to 0.4598	No	ns	0.2903
Inoculum vs. 1 hr	0.4446	0.1748 to 0.7144	Yes	***	0.0002
Inoculum vs. 2 hr	0.5279	0.2581 to 0.7976	Yes	****	<0.0001
Inoculum vs. 20 hr	0.3101	0.04033 to 0.5799	Yes	*	0.0162
15 min vs. 1 hr	0.2546	-0.01522 to 0.5244	No	ns	0.0736
15 min vs. 2 hr	0.3379	0.06807 to 0.6076	Yes	**	0.0069
15 min vs. 20 hr	0.1201	-0.1497 to 0.3899	No	ns	0.7241
1 hr vs. 2 hr	0.08329	-0.1865 to 0.3531	No	ns	0.9088
1 hr vs. 20 hr	-0.1344	-0.4042 to 0.1353	No	ns	0.6326
2 hr vs. 20 hr	-0.2177	-0.4875 to 0.05205	No	ns	0.1704

WT Llo + WT Lpn					
Inoculum vs. 15 min	0.2936	0.02386 to 0.5634	Yes	*	0.0261
Inoculum vs. 1 hr	0.4736	0.2038 to 0.7434	Yes	****	<0.0001
Inoculum vs. 2 hr	0.5055	0.2358 to 0.7753	Yes	****	<0.0001
Inoculum vs. 20 hr	0.4666	0.1968 to 0.7363	Yes	****	<0.0001
15 min vs. 1 hr	0.18	-0.08982 to 0.4498	No	ns	0.3441
15 min vs. 2 hr	0.2119	-0.05789 to 0.4817	No	ns	0.1921
15 min vs. 20 hr	0.1729	-0.09688 to 0.4427	No	ns	0.3849
1 hr vs. 2 hr	0.03193	-0.2379 to 0.3017	No	ns	0.9973
1 hr vs. 20 hr	-0.00706	-0.2769 to 0.2627	No	ns	>0.9999
2 hr vs. 20 hr	-0.03899	-0.3088 to 0.2308	No	ns	0.9942
ΔT4SS Llo + WT Lpn					
Inoculum vs. 15 min	0.3158	0.04604 to 0.5856	Yes	*	0.0137
Inoculum vs. 1 hr	0.4219	0.1521 to 0.6916	Yes	***	0.0004
Inoculum vs. 2 hr	0.5281	0.2583 to 0.7979	Yes	****	<0.0001
Inoculum vs. 20 hr	0.4865	0.2167 to 0.7562	Yes	****	<0.0001
15 min vs. 1 hr	0.106	-0.1638 to 0.3758	No	ns	0.8057
15 min vs. 2 hr	0.2123	-0.05751 to 0.4821	No	ns	0.1906
15 min vs. 20 hr	0.1706	-0.09916 to 0.4404	No	ns	0.3985
1 hr vs. 2 hr	0.1063	-0.1635 to 0.3760	No	ns	0.8045
1 hr vs. 20 hr	0.06461	-0.2052 to 0.3344	No	ns	0.9621
2 hr vs. 20 hr	-0.04165	-0.3114 to 0.2281	No	ns	0.9926
WT Lpn					
Inoculum vs. 15 min	0	-0.2698 to 0.2698	No	ns	>0.9999
Inoculum vs. 1 hr	0.5501	0.2803 to 0.8198	Yes	****	<0.0001
Inoculum vs. 2 hr	0.9127	0.6429 to 1.182	Yes	****	<0.0001
Inoculum vs. 20 hr	1.878	1.609 to 2.148	Yes	****	<0.0001
15 min vs. 1 hr	0.5501	0.2803 to 0.8198	Yes	****	<0.0001
15 min vs. 2 hr	0.9127	0.6429 to 1.182	Yes	****	<0.0001
15 min vs. 20 hr	1.878	1.609 to 2.148	Yes	****	<0.0001
1 hr vs. 2 hr	0.3626	0.09285 to 0.6324	Yes	**	0.0031
1 hr vs. 20 hr	1.328	1.058 to 1.598	Yes	****	<0.0001
2 hr vs. 20 hr	0.9656	0.6958 to 1.235	Yes	****	<0.0001

WT Llo + WT Lpn					
Inoculum vs. 15 min	0.3448	0.07500 to 0.6146	Yes	**	0.0055
Inoculum vs. 1 hr	0.4342	0.1645 to 0.7040	Yes	***	0.0002
Inoculum vs. 2 hr	0.8488	0.5790 to 1.119	Yes	****	<0.0001
Inoculum vs. 20 hr	1.809	1.539 to 2.078	Yes	****	<0.0001
15 min vs. 1 hr	0.08946	-0.1803 to 0.3592	No	ns	0.8849
15 min vs. 2 hr	0.504	0.2342 to 0.7738	Yes	****	<0.0001
15 min vs. 20 hr	1.464	1.194 to 1.734	Yes	****	<0.0001
1 hr vs. 2 hr	0.4145	0.1447 to 0.6843	Yes	***	0.0005
1 hr vs. 20 hr	1.374	1.105 to 1.644	Yes	****	<0.0001
2 hr vs. 20 hr	0.9599	0.6901 to 1.230	Yes	****	<0.0001
ΔT4SS Llo + WT Lpn					
Inoculum vs. 15 min	0.1983	-0.07151 to 0.4681	No	ns	0.25
Inoculum vs. 1 hr	0.4912	0.2214 to 0.7610	Yes	****	<0.0001
Inoculum vs. 2 hr	0.9798	0.7100 to 1.250	Yes	****	<0.0001
Inoculum vs. 20 hr	1.809	1.540 to 2.079	Yes	****	<0.0001
15 min vs. 1 hr	0.2929	0.02310 to 0.5627	Yes	*	0.0267
15 min vs. 2 hr	0.7815	0.5117 to 1.051	Yes	****	<0.0001
15 min vs. 20 hr	1.611	1.341 to 1.881	Yes	****	<0.0001
1 hr vs. 2 hr	0.4886	0.2188 to 0.7584	Yes	****	<0.0001
1 hr vs. 20 hr	1.318	1.049 to 1.588	Yes	****	<0.0001
2 hr vs. 20 hr	0.8297	0.5599 to 1.100	Yes	****	<0.0001

Test details	Mean 1	Mean 2	Mean Diff.	SE of diff.	N1	N2	q	DF
Inoculum								
WT Llo vs. ΔT4SS Llo	5.423	5.328	0.09436	0.09635	3	3	1.385	70
WT Llo vs. WT Llo + WT Lpn	5.423	5.367	0.05532	0.09635	3	3	0.8121	70
WT Llo vs. ΔT4SS Llo + WT Lpn	5.423	5.465	-0.04261	0.09635	3	3	0.6254	70
WT Llo vs. WT Lpn	5.423	5.346	0.07624	0.09635	3	3	1.119	70
WT Llo vs. WT Llo + WT Lpn	5.423	5.335	0.08763	0.09635	3	3	1.286	70
WT Llo vs. ΔT4SS Llo + WT Lpn	5.423	5.285	0.1381	0.09635	3	3	2.027	70
ΔT4SS Llo vs. WT Llo + WT Lpn	5.328	5.367	-0.03904	0.09635	3	3	0.573	70
ΔT4SS Llo vs. ΔT4SS Llo + WT Lpn	5.328	5.465	-0.137	0.09635	3	3	2.01	70
ΔT4SS Llo vs. WT Lpn	5.328	5.346	-0.01812	0.09635	3	3	0.266	70

ΔT4SS Llo vs. WT Llo + WT Lpn	5.328	5.335	-0.00673	0.09635	3	3	0.09885	70
ΔT4SS Llo vs. ΔT4SS Llo + WT Lpn	5.328	5.285	0.04376	0.09635	3	3	0.6423	70
WT Llo + WT Lpn vs. ΔT4SS Llo + WT Lpn	5.367	5.465	-0.09793	0.09635	3	3	1.437	70
WT Llo + WT Lpn vs. WT Lpn	5.367	5.346	0.02092	0.09635	3	3	0.307	70
WT Llo + WT Lpn vs. WT Llo + WT Lpn	5.367	5.335	0.0323	0.09635	3	3	0.4742	70
WT Llo + WT Lpn vs. ΔT4SS Llo + WT Lpn	5.367	5.285	0.0828	0.09635	3	3	1.215	70
ΔT4SS Llo + WT Lpn vs. WT Lpn	5.465	5.346	0.1189	0.09635	3	3	1.745	70
ΔT4SS Llo + WT Lpn vs. WT Llo + WT Lpn	5.465	5.335	0.1302	0.09635	3	3	1.912	70
ΔT4SS Llo + WT Lpn vs. ΔT4SS Llo + WT Lpn	5.465	5.285	0.1807	0.09635	3	3	2.653	70
WT Lpn vs. WT Llo + WT Lpn	5.346	5.335	0.01138	0.09635	3	3	0.1671	70
WT Lpn vs. ΔT4SS Llo + WT Lpn	5.346	5.285	0.06188	0.09635	3	3	0.9083	70
WT Llo + WT Lpn vs. ΔT4SS Llo + WT Lpn	5.335	5.285	0.05049	0.09635	3	3	0.7412	70
15 min								
WT Llo vs. ΔT4SS Llo	5.121	5.138	-0.01712	0.09635	3	3	0.2513	70
WT Llo vs. WT Llo + WT Lpn	5.121	5.074	0.04749	0.09635	3	3	0.697	70
WT Llo vs. ΔT4SS Llo + WT Lpn	5.121	5.149	-0.02826	0.09635	3	3	0.4148	70
WT Llo vs. WT Lpn	5.121	5.346	-0.2252	0.09635	3	3	3.306	70
WT Llo vs. WT Llo + WT Lpn	5.121	4.99	0.1309	0.09635	3	3	1.922	70
WT Llo vs. ΔT4SS Llo + WT Lpn	5.121	5.086	0.03491	0.09635	3	3	0.5124	70
ΔT4SS Llo vs. WT Llo + WT Lpn	5.138	5.074	0.06461	0.09635	3	3	0.9483	70
ΔT4SS Llo vs. ΔT4SS Llo + WT Lpn	5.138	5.149	-0.01114	0.09635	3	3	0.1635	70
ΔT4SS Llo vs. WT Lpn	5.138	5.346	-0.2081	0.09635	3	3	3.055	70
ΔT4SS Llo vs. WT Llo + WT Lpn	5.138	4.99	0.1481	0.09635	3	3	2.173	70
ΔT4SS Llo vs. ΔT4SS Llo + WT Lpn	5.138	5.086	0.05203	0.09635	3	3	0.7637	70
WT Llo + WT Lpn vs. ΔT4SS Llo + WT Lpn	5.074	5.149	-0.07575	0.09635	3	3	1.112	70
WT Llo + WT Lpn vs. WT Lpn	5.074	5.346	-0.2727	0.09635	3	3	4.003	70
WT Llo + WT Lpn vs. WT Llo + WT Lpn	5.074	4.99	0.08345	0.09635	3	3	1.225	70
WT Llo + WT Lpn vs. ΔT4SS Llo + WT Lpn	5.074	5.086	-0.01257	0.09635	3	3	0.1846	70
ΔT4SS Llo + WT Lpn vs. WT Lpn	5.149	5.346	-0.197	0.09635	3	3	2.891	70
ΔT4SS Llo + WT Lpn vs. WT Llo + WT Lpn	5.149	4.99	0.1592	0.09635	3	3	2.337	70
ΔT4SS Llo + WT Lpn vs. ΔT4SS Llo + WT Lpn	5.149	5.086	0.06317	0.09635	3	3	0.9273	70
WT Lpn vs. WT Llo + WT Lpn	5.346	4.99	0.3562	0.09635	3	3	5.228	70
WT Lpn vs. ΔT4SS Llo + WT Lpn	5.346	5.086	0.2602	0.09635	3	3	3.819	70
WT Llo + WT Lpn vs. ΔT4SS Llo + WT Lpn	4.99	5.086	-0.09602	0.09635	3	3	1.409	70
1 hr								

WT Llo vs. ΔT4SS Llo	4.916	4.884	0.0323	0.09635	3	3	0.4742	70
WT Llo vs. WT Llo + WT Lpn	4.916	4.894	0.02232	0.09635	3	3	0.3276	70
WT Llo vs. ΔT4SS Llo + WT Lpn	4.916	5.043	-0.1274	0.09635	3	3	1.87	70
WT Llo vs. WT Lpn	4.916	4.796	0.1197	0.09635	3	3	1.757	70
WT Llo vs. WT Llo + WT Lpn	4.916	4.901	0.01525	0.09635	3	3	0.2239	70
WT Llo vs. ΔT4SS Llo + WT Lpn	4.916	4.793	0.1227	0.09635	3	3	1.8	70
ΔT4SS Llo vs. WT Llo + WT Lpn	4.884	4.894	-0.00999	0.09635	3	3	0.1466	70
ΔT4SS Llo vs. ΔT4SS Llo + WT Lpn	4.884	5.043	-0.1597	0.09635	3	3	2.344	70
ΔT4SS Llo vs. WT Lpn	4.884	4.796	0.08737	0.09635	3	3	1.282	70
ΔT4SS Llo vs. WT Llo + WT Lpn	4.884	4.901	-0.01705	0.09635	3	3	0.2503	70
ΔT4SS Llo vs. ΔT4SS Llo + WT Lpn	4.884	4.793	0.09036	0.09635	3	3	1.326	70
WT Llo + WT Lpn vs. ΔT4SS Llo + WT Lpn	4.894	5.043	-0.1497	0.09635	3	3	2.197	70
WT Llo + WT Lpn vs. WT Lpn	4.894	4.796	0.09736	0.09635	3	3	1.429	70
WT Llo + WT Lpn vs. WT Llo + WT Lpn	4.894	4.901	-0.00706	0.09635	3	3	0.1037	70
WT Llo + WT Lpn vs. ΔT4SS Llo + WT Lpn	4.894	4.793	0.1003	0.09635	3	3	1.473	70
ΔT4SS Llo + WT Lpn vs. WT Lpn	5.043	4.796	0.2471	0.09635	3	3	3.626	70
ΔT4SS Llo + WT Lpn vs. WT Llo + WT Lpn	5.043	4.901	0.1426	0.09635	3	3	2.094	70
ΔT4SS Llo + WT Lpn vs. ΔT4SS Llo + WT Lpn	5.043	4.793	0.25	0.09635	3	3	3.67	70
WT Lpn vs. WT Llo + WT Lpn	4.796	4.901	-0.1044	0.09635	3	3	1.533	70
WT Lpn vs. ΔT4SS Llo + WT Lpn	4.796	4.793	0.002985	0.09635	3	3	0.04381	70
WT Llo + WT Lpn vs. ΔT4SS Llo + WT Lpn	4.901	4.793	0.1074	0.09635	3	3	1.577	70
2 hr								
WT Llo vs. ΔT4SS Llo	4.796	4.8	-0.00408	0.09635	3	3	0.05986	70
WT Llo vs. WT Llo + WT Lpn	4.796	4.862	-0.06543	0.09635	3	3	0.9604	70
WT Llo vs. ΔT4SS Llo + WT Lpn	4.796	4.937	-0.1408	0.09635	3	3	2.067	70
WT Llo vs. WT Lpn	4.796	4.434	0.3626	0.09635	3	3	5.323	70
WT Llo vs. WT Llo + WT Lpn	4.796	4.486	0.3101	0.09635	3	3	4.552	70
WT Llo vs. ΔT4SS Llo + WT Lpn	4.796	4.305	0.4916	0.09635	3	3	7.216	70
ΔT4SS Llo vs. WT Llo + WT Lpn	4.8	4.862	-0.06135	0.09635	3	3	0.9006	70
ΔT4SS Llo vs. ΔT4SS Llo + WT Lpn	4.8	4.937	-0.1367	0.09635	3	3	2.007	70
ΔT4SS Llo vs. WT Lpn	4.8	4.434	0.3667	0.09635	3	3	5.383	70
ΔT4SS Llo vs. WT Llo + WT Lpn	4.8	4.486	0.3142	0.09635	3	3	4.611	70
ΔT4SS Llo vs. ΔT4SS Llo + WT Lpn	4.8	4.305	0.4957	0.09635	3	3	7.276	70
WT Llo + WT Lpn vs. ΔT4SS Llo + WT Lpn	4.862	4.937	-0.07537	0.09635	3	3	1.106	70
WT Llo + WT Lpn vs. WT Lpn	4.862	4.434	0.4281	0.09635	3	3	6.283	70
WT Llo + WT Lpn vs. WT Llo + WT Lpn	4.862	4.486	0.3755	0.09635	3	3	5.512	70

WT Llo + WT Lpn vs. ΔT4SS Llo + WT Lpn	4.862	4.305	0.557	0.09635	3	3	8.176	70
ΔT4SS Llo + WT Lpn vs. WT Lpn	4.937	4.434	0.5034	0.09635	3	3	7.39	70
ΔT4SS Llo + WT Lpn vs. WT Llo + WT Lpn	4.937	4.486	0.4509	0.09635	3	3	6.618	70
ΔT4SS Llo + WT Lpn vs. ΔT4SS Llo + WT Lpn	4.937	4.305	0.6324	0.09635	3	3	9.282	70
WT Lpn vs. WT Llo + WT Lpn	4.434	4.486	-0.05255	0.09635	3	3	0.7714	70
WT Lpn vs. ΔT4SS Llo + WT Lpn	4.434	4.305	0.129	0.09635	3	3	1.893	70
WT Llo + WT Lpn vs. ΔT4SS Llo + WT Lpn	4.486	4.305	0.1815	0.09635	3	3	2.664	70
20 hr								
WT Llo vs. ΔT4SS Llo	4.859	5.018	-0.159	0.09635	3	3	2.334	70
WT Llo vs. WT Llo + WT Lpn	4.859	4.901	-0.04165	0.09635	3	3	0.6113	70
WT Llo vs. ΔT4SS Llo + WT Lpn	4.859	4.979	-0.1197	0.09635	3	3	1.757	70
WT Llo vs. WT Lpn	4.859	3.468	1.391	0.09635	3	3	20.42	70
WT Llo vs. WT Llo + WT Lpn	4.859	3.526	1.333	0.09635	3	3	19.56	70
WT Llo vs. ΔT4SS Llo + WT Lpn	4.859	3.475	1.384	0.09635	3	3	20.32	70
ΔT4SS Llo vs. WT Llo + WT Lpn	5.018	4.901	0.1174	0.09635	3	3	1.723	70
ΔT4SS Llo vs. ΔT4SS Llo + WT Lpn	5.018	4.979	0.03937	0.09635	3	3	0.5778	70
ΔT4SS Llo vs. WT Lpn	5.018	3.468	1.55	0.09635	3	3	22.75	70
ΔT4SS Llo vs. WT Llo + WT Lpn	5.018	3.526	1.492	0.09635	3	3	21.9	70
ΔT4SS Llo vs. ΔT4SS Llo + WT Lpn	5.018	3.475	1.543	0.09635	3	3	22.65	70
WT Llo + WT Lpn vs. ΔT4SS Llo + WT Lpn	4.901	4.979	-0.07803	0.09635	3	3	1.145	70
WT Llo + WT Lpn vs. WT Lpn	4.901	3.468	1.433	0.09635	3	3	21.03	70
WT Llo + WT Lpn vs. WT Llo + WT Lpn	4.901	3.526	1.374	0.09635	3	3	20.17	70
WT Llo + WT Lpn vs. ΔT4SS Llo + WT Lpn	4.901	3.475	1.426	0.09635	3	3	20.93	70
ΔT4SS Llo + WT Lpn vs. WT Lpn	4.979	3.468	1.511	0.09635	3	3	22.17	70
ΔT4SS Llo + WT Lpn vs. WT Llo + WT Lpn	4.979	3.526	1.452	0.09635	3	3	21.32	70
ΔT4SS Llo + WT Lpn vs. ΔT4SS Llo + WT Lpn	4.979	3.475	1.504	0.09635	3	3	22.07	70
WT Lpn vs. WT Llo + WT Lpn	3.468	3.526	-0.05825	0.09635	3	3	0.8549	70
WT Lpn vs. ΔT4SS Llo + WT Lpn	3.468	3.475	-0.00695	0.09635	3	3	0.1019	70
WT Llo + WT Lpn vs. ΔT4SS Llo + WT Lpn	3.526	3.475	0.0513	0.09635	3	3	0.753	70
WT Llo								
Inoculum vs. 15 min	5.423	5.121	0.3015	0.09635	3	3	4.425	70
Inoculum vs. 1 hr	5.423	4.916	0.5066	0.09635	3	3	7.436	70
Inoculum vs. 2 hr	5.423	4.796	0.6263	0.09635	3	3	9.193	70
Inoculum vs. 20 hr	5.423	4.859	0.5635	0.09635	3	3	8.272	70
15 min vs. 1 hr	5.121	4.916	0.2051	0.09635	3	3	3.011	70

15 min vs. 2 hr	5.121	4.796	0.3248	0.09635	3	3	4.768	70
15 min vs. 20 hr	5.121	4.859	0.262	0.09635	3	3	3.846	70
1 hr vs. 2 hr	4.916	4.796	0.1197	0.09635	3	3	1.757	70
1 hr vs. 20 hr	4.916	4.859	0.0569	0.09635	3	3	0.8352	70
2 hr vs. 20 hr	4.796	4.859	-0.06278	0.09635	3	3	0.9214	70
ΔT4SS Llo								
Inoculum vs. 15 min	5.328	5.138	0.19	0.09635	3	3	2.789	70
Inoculum vs. 1 hr	5.328	4.884	0.4446	0.09635	3	3	6.525	70
Inoculum vs. 2 hr	5.328	4.8	0.5279	0.09635	3	3	7.748	70
Inoculum vs. 20 hr	5.328	5.018	0.3101	0.09635	3	3	4.552	70
15 min vs. 1 hr	5.138	4.884	0.2546	0.09635	3	3	3.737	70
15 min vs. 2 hr	5.138	4.8	0.3379	0.09635	3	3	4.959	70
15 min vs. 20 hr	5.138	5.018	0.1201	0.09635	3	3	1.763	70
1 hr vs. 2 hr	4.884	4.8	0.08329	0.09635	3	3	1.223	70
1 hr vs. 20 hr	4.884	5.018	-0.1344	0.09635	3	3	1.973	70
2 hr vs. 20 hr	4.8	5.018	-0.2177	0.09635	3	3	3.196	70
WT Llo + WT Lpn								
Inoculum vs. 15 min	5.367	5.074	0.2936	0.09635	3	3	4.31	70
Inoculum vs. 1 hr	5.367	4.894	0.4736	0.09635	3	3	6.952	70
Inoculum vs. 2 hr	5.367	4.862	0.5055	0.09635	3	3	7.42	70
Inoculum vs. 20 hr	5.367	4.901	0.4666	0.09635	3	3	6.848	70
15 min vs. 1 hr	5.074	4.894	0.18	0.09635	3	3	2.642	70
15 min vs. 2 hr	5.074	4.862	0.2119	0.09635	3	3	3.11	70
15 min vs. 20 hr	5.074	4.901	0.1729	0.09635	3	3	2.538	70
1 hr vs. 2 hr	4.894	4.862	0.03193	0.09635	3	3	0.4686	70
1 hr vs. 20 hr	4.894	4.901	-0.00706	0.09635	3	3	0.1037	70
2 hr vs. 20 hr	4.862	4.901	-0.03899	0.09635	3	3	0.5723	70
ΔT4SS Llo + WT Lpn								
Inoculum vs. 15 min	5.465	5.149	0.3158	0.09635	3	3	4.636	70
Inoculum vs. 1 hr	5.465	5.043	0.4219	0.09635	3	3	6.192	70
Inoculum vs. 2 hr	5.465	4.937	0.5281	0.09635	3	3	7.752	70
Inoculum vs. 20 hr	5.465	4.979	0.4865	0.09635	3	3	7.14	70
15 min vs. 1 hr	5.149	5.043	0.106	0.09635	3	3	1.556	70
15 min vs. 2 hr	5.149	4.937	0.2123	0.09635	3	3	3.116	70

15 min vs. 20 hr	5.149	4.979	0.1706	0.09635	3	3	2.505	70
1 hr vs. 2 hr	5.043	4.937	0.1063	0.09635	3	3	1.56	70
1 hr vs. 20 hr	5.043	4.979	0.06461	0.09635	3	3	0.9483	70
2 hr vs. 20 hr	4.937	4.979	-0.04165	0.09635	3	3	0.6113	70
WT Lpn								
Inoculum vs. 15 min	5.346	5.346	0	0.09635	3	3	0	70
Inoculum vs. 1 hr	5.346	4.796	0.5501	0.09635	3	3	8.074	70
Inoculum vs. 2 hr	5.346	4.434	0.9127	0.09635	3	3	13.4	70
Inoculum vs. 20 hr	5.346	3.468	1.878	0.09635	3	3	27.57	70
15 min vs. 1 hr	5.346	4.796	0.5501	0.09635	3	3	8.074	70
15 min vs. 2 hr	5.346	4.434	0.9127	0.09635	3	3	13.4	70
15 min vs. 20 hr	5.346	3.468	1.878	0.09635	3	3	27.57	70
1 hr vs. 2 hr	4.796	4.434	0.3626	0.09635	3	3	5.323	70
1 hr vs. 20 hr	4.796	3.468	1.328	0.09635	3	3	19.5	70
2 hr vs. 20 hr	4.434	3.468	0.9656	0.09635	3	3	14.17	70
WT Llo + WT Lpn								
Inoculum vs. 15 min	5.335	4.99	0.3448	0.09635	3	3	5.061	70
Inoculum vs. 1 hr	5.335	4.901	0.4342	0.09635	3	3	6.374	70
Inoculum vs. 2 hr	5.335	4.486	0.8488	0.09635	3	3	12.46	70
Inoculum vs. 20 hr	5.335	3.526	1.809	0.09635	3	3	26.55	70
15 min vs. 1 hr	4.99	4.901	0.08946	0.09635	3	3	1.313	70
15 min vs. 2 hr	4.99	4.486	0.504	0.09635	3	3	7.397	70
15 min vs. 20 hr	4.99	3.526	1.464	0.09635	3	3	21.49	70
1 hr vs. 2 hr	4.901	4.486	0.4145	0.09635	3	3	6.084	70
1 hr vs. 20 hr	4.901	3.526	1.374	0.09635	3	3	20.17	70
2 hr vs. 20 hr	4.486	3.526	0.9599	0.09635	3	3	14.09	70
ΔT4SS Llo + WT Lpn								
Inoculum vs. 15 min	5.285	5.086	0.1983	0.09635	3	3	2.91	70
Inoculum vs. 1 hr	5.285	4.793	0.4912	0.09635	3	3	7.209	70
Inoculum vs. 2 hr	5.285	4.305	0.9798	0.09635	3	3	14.38	70
Inoculum vs. 20 hr	5.285	3.475	1.809	0.09635	3	3	26.56	70
15 min vs. 1 hr	5.086	4.793	0.2929	0.09635	3	3	4.299	70
15 min vs. 2 hr	5.086	4.305	0.7815	0.09635	3	3	11.47	70
15 min vs. 20 hr	5.086	3.475	1.611	0.09635	3	3	23.65	70

1 hr vs. 2 hr	4.793	4.305	0.4886	0.09635	3	3	7.172	70
1 hr vs. 20 hr	4.793	3.475	1.318	0.09635	3	3	19.35	70
2 hr vs. 20 hr	4.305	3.475	0.8297	0.09635	3	3		

Supplemental Figure 1. Impact of opsonization and growth phase on *L. longbeachae* survival within neutrophils. To determine if opsonization enhanced neutrophil uptake or degradation of *Llo*, cells were infected with un-opsonized or human serum opsonized *Llo* for 15 min. (A) Representative confocal images of neutrophils infected with WT *Llo* or Δ*T4 Llo* at 15 min post-infection. Extracellular (red) and intracellular (green) bacteria were differentially labeled using antibodies. Cell nuclei were stained with DAPI (blue). (B) Uptake of WT and Δ*T4 Llo* was assessed at 15 minutes post-infection. Data shown are percentage of neutrophils with internal bacteria ± SD, n = 3. (C) Degradation of *Llo* was examined by confocal microscopy at 15 min post-infection. Data are shown as mean percent killed and degraded bacteria ± SD, n = 3. (D) To determine survival of *Llo* grown *in vitro* to exponential or post-exponential phase, infected neutrophils were lysed at 5-, 15-, 60-, and 120-min post-infection. CFUs were determined by serial dilutions on BCYE agar plates ± SD, n = 3. Data shown are representative of three independent biological repeats.

Supplemental Figure 2. *Trans*-acting Inhibition of neutrophil microbicidal activities by *L. longbeachae*. Representative confocal images of neutrophils solo-infected (A) or co-infected (B) for 15 minutes with WT *Llo* NSW150, WT *Llo* D4968, WT *Lpn*, or Δ*T4 Lpn* strains as indicated. (C) Bacterial degradation was determined by confocal microscopy. Data are shown as mean percent killed and degraded D4968 *Llo* (green), NSW150 *Llo* (red), or *Lpn* (blue) ± SD, n = 3 (scatter dot plot). Data shown are representative of three independent biological repeats.

Supplemental Figure 3. Super-infections of neutrophils with *L. longbeachae* and Δ*T4 L. pneumophila*. (A) Experimental design for super-infections of neutrophils. Super-infected cells were

first subjected to primary infections (1°) individually with *Llo* or *Lpn* strains for 15 min, then subjected to secondary infections (2°) with the other bacterial species for another 15 min. Control cells were solo-infected with *Llo* or *Lpn* at the same time as secondary infections for 15 min. (B-D) Neutrophils were solo-infected with WT or Δ*T4* bacterial strains or subjected to 15 min primary infections (1°) followed by super-infection (2°) for an additional 15 minutes. Bacterial morphology was examined by confocal microscopy in neutrophils solo-infected (B) or super-infected (C). Bacteria were labeled with anti-*Llo* (red) or anti-*Lpn* (green) antibodies. Cell nuclei were stained with DAPI (blue). (D) Data are shown as mean percent killed and degraded *Llo* (red) or *Lpn* (blue) ± SD, n = 3 (scatter plot dots). Data shown are representative of three independent biological replicates.

Supplemental Figure 4. Uptake and degradation of bacteria in solo- and dual-infected cells during super-infections. Quantification of bacterial uptake and degradation for solo- and dual-infected cells during super-infections. Uptake of *Llo* (A) and *Lpn* (B) strains was assessed following super-infections. Data are shown as percentage of neutrophils with internal bacteria as indicated for cells solo-infected with *Llo* strains (red), solo-infected with *Lpn* (blue) strains, or dual-infected (purple) ± SD, n = 3. Degradation of *Llo* (C) and *Lpn* (D) strains was assessed following super-infections. Data are shown as mean percent killed and degraded bacteria as indicated for cells solo-infected with *Llo* strains (red), solo-infected with *Lpn* (blue) strains, or dual-infected (purple) ± SD, n = 3. Data shown are representative of three independent biological replicates.

Supplemental Figure 5. Enumeration of viable *L. longbeachae* and *L. pneumophila* during co-infections. CFU experiments were designed to determine if protection and increased survival of *Lpn* by *Llo* could be observed without single cell analysis. To select for individual bacterial strains, WT

and Δ*T4 Lpn* strains were transformed with a chloramphenicol resistance plasmid while WT and Δ*T4 Llo* strains were transformed with a kanamycin resistance plasmid. (A) Bacteria were grown overnight in BYE broth with antibiotics prior to infection. Solo- or co-infected neutrophils were lysed at 0-, 0.25-, 1-, 2-, and 20 hrs post-infection. Serial dilutions of lysates were plated on BCYE agar with kanamycin or chloramphenicol to select for and quantify CFUs of *Llo* or *Lpn* strains, respectively ± SD, n = 3. Data shown are representative of three independent biological replicates.

Supplemental Figure 6. Exclusion of the CD66b specific granule membrane marker from the *L. longbeachae* phagosome. To further determine fusion of specific granules to bacterial phagosomes, neutrophils were labeled for the membrane marker CD66b. Neutrophils were solo-infected with WT or Δ*T4* bacterial strains for 15 min or subjected to 15 min primary infections (1°) followed by super-infection (2°) for an additional 15 minutes. Representative confocal images of specific granule fusion to phagosomes during solo-infections (A) or super-infections (B). Bacteria were labeled with anti-*Llo* antibody (cyan) or anti-*Lpn* antibody (green). Zymosan A BioParticlesTM Alexa Fluor 594 are shown as green. Specific granules were labeled with anti-CD66b antibody (red). White arrows indicate positive co-localization of CD66b with pathogen-containing phagosomes. Data are shown as mean percent co-localization of CD66b to *Llo* phagosomes (red) or *Lpn* phagosomes (blue) during infections. Zymosan was included as a positive control (green) ± SD, n = 3 (scatter plot dots). Data shown are representative of three independent biological repeats.

Supplemental Figure 7. Degradation of UV-Killed *L. longbeachae* by human neutrophils. Neutrophils were infected with viable WT, Δ*T4*, formalin-killed (FK), heat-killed (HK), or UV-killed (UVK) *Llo* and *Lpn*. (A and D) Representative confocal images of neutrophils infected with viable or

killed strains of *Llo* (A) or *Lpn* (D) at 1 hr post-infection. Bacteria were labeled with anti-*Llo* antibody (red) or anti-*Lpn* antibody (green). Cell nuclei were stained with DAPI (blue). (B and E) Uptake of viable and killed *Llo* (B) or *Lpn* (E) was determined at 1 hr post-infection. Data are shown as percentage of neutrophils with internal *Llo* strains (red) or *Lpn* (blue) strains ± SD, n = 3. (C and F) Bacterial degradation was examined by confocal microscopy at 1 hr post-infection for *Llo* (C) or *Lpn* (F). Data are shown as mean percent killed and degraded *Llo* (red) or *Lpn* (blue) ± SD, n = 3. Data shown are representative of three independent biological repeats.

Supplemental Figure 8. Failure of robust degranulation of specific granules by neutrophils in response to *L. longbeachae*. (A-I) Neutrophils were infected with WT, Δ*T4*, or UV-killed (UVK) *Legionella* strains for up to 20 hrs at an MOI of 5. Cells were stimulated with fMLP or infected with E. coli at an MOI of 5 as additional positive controls. Aliquots of cell supernatants were collected at 0.5-, 1-, 4-, 6-, 12- and 20 hrs post-infection to quantify release of specific granules using lipocalin as a marker by ELISA. Data are shown as kinetic graphs of lipocalin release during 20 hrs post-infection for indicated groups ± SEM, n = 3. Data shown are representative of three independent biological repeats.

Supplemental Figure 9. Failure of robust degranulation of azurophilic granules by neutrophils in response to *L. longbeachae*. (A-I) Neutrophils were infected with WT, Δ*T4*, or UV-killed (UVK) *Legionella* strains for up to 20 hrs at an MOI of 5. Cells were stimulated with fMLP or infected with E. coli at an MOI of 5 as additional positive controls. Aliquots of cell supernatants were collected at 0.5-, 1-, 4-, 6-, 12- and 20 hrs post-infection to quantify release of azurophilic granules using elastase as a marker by ELISA. Data are shown as kinetic graphs of elastase release during 20 hrs post-infection

for indicated groups ± SEM, n = 3. Data shown are representative of three independent biological repeats.

Supplemental Figure 10. Failure of robust IL-8 production by neutrophils in response to *L. longbeachae*. (A-H) Neutrophils were infected with WT, Δ*T4*, or UV-killed (UVK) *Legionella* strains for up to 20 hrs at an MOI of 5. Cells were infected with E. coli at an MOI of 5 as a positive control. Aliquots of cell supernatants were collected at 0.5-, 1-, 4-, 6-, 12- and 20 hrs post-infection to quantify production of IL-8 by ELISA. Data are shown as kinetic graphs of IL-8 production during 20 hrs post-infection for indicated groups ± SEM, n = 3. Data shown are representative of three independent biological repeats.

Figure S1

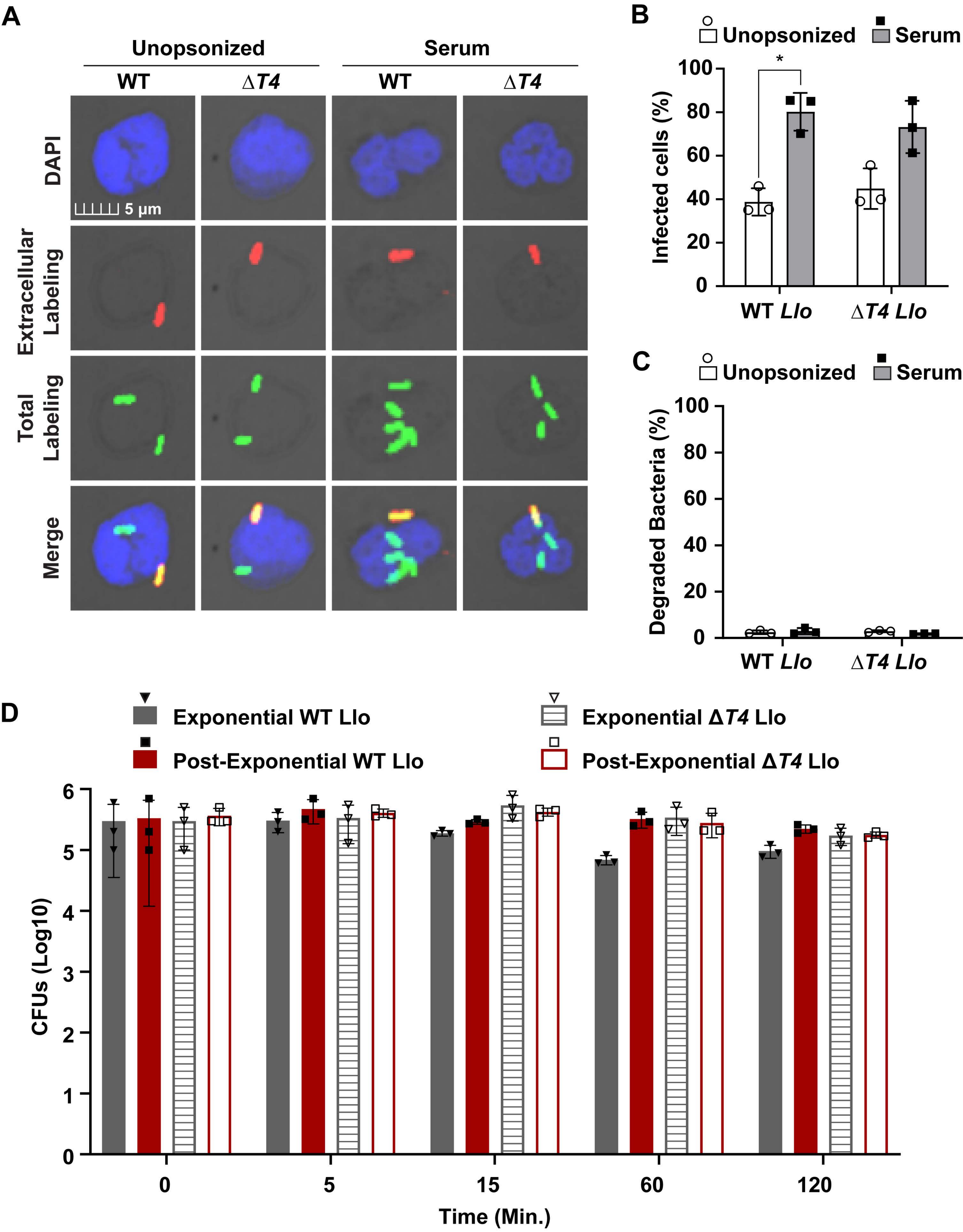

A
Unopsonized
Serum
WT
ΔT4
WT
ΔT4
DAPI
5 µm
Extracellular Labeling
Total Labeling
Merge
B
Unopsonized
Serum
Infected cells (%)
100
80
60
40
20
0
*
WT Llo
ΔT4 Llo
C
Unopsonized
Serum
Degraded Bacteria (%)
100
80
60
40
20
0
WT Llo
ΔT4 Llo
D
Exponential WT Llo
Exponential ΔT4 Llo
Post-Exponential WT Llo
Post-Exponential ΔT4 Llo
CFUs (Log10)
6
5
4
3
2
1
0
0
5
15
60
120
Time (Min.)

Figure S2

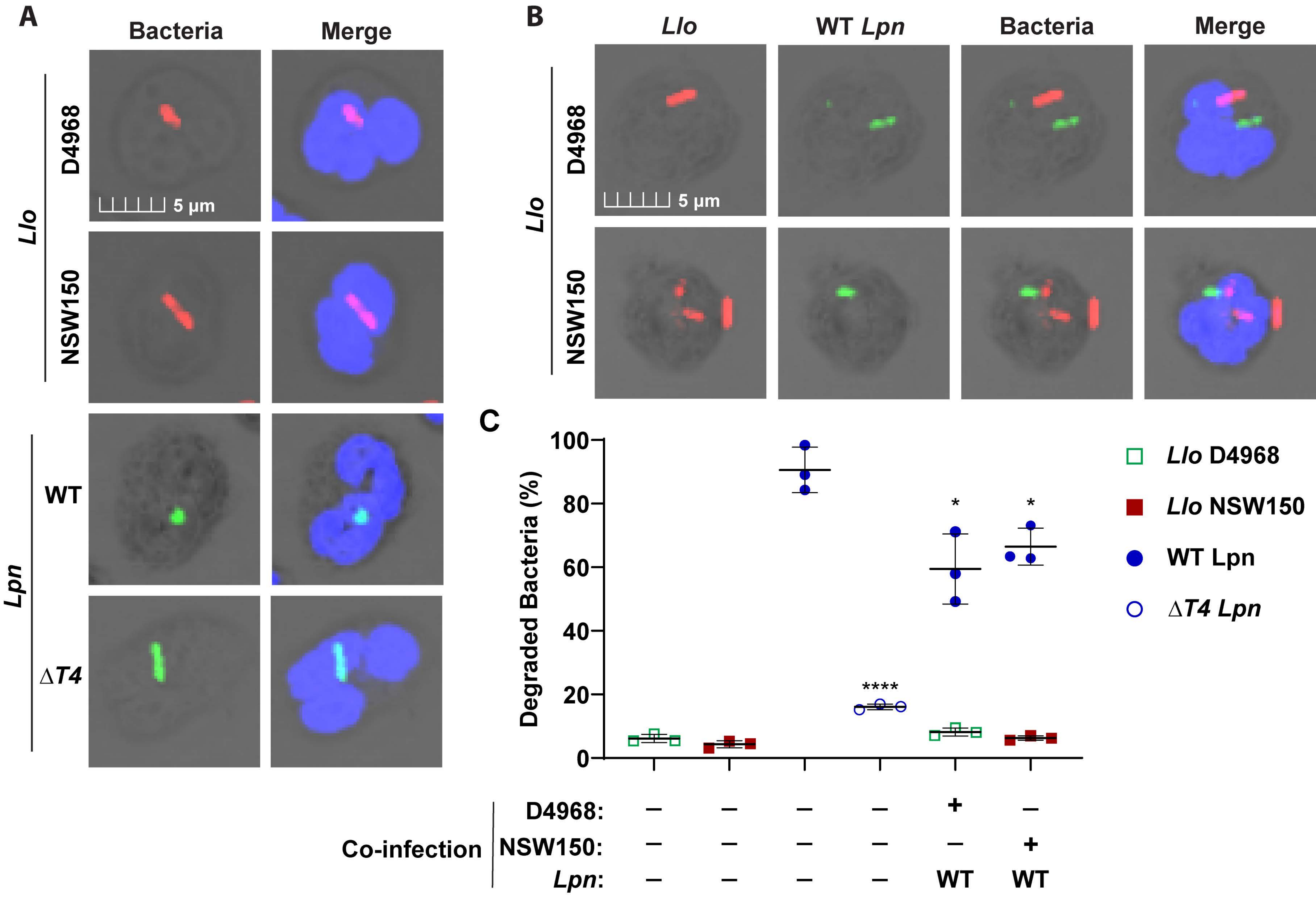

A
Bacteria
Merge
Llo
D4968
NSW150
5 µm
Lpn
WT
ΔT4
B
Llo
WT Lpn
Bacteria
Merge
D4968
NSW150
5 µm
C
Degraded Bacteria (%)
100
80
60
40
20
0
****
*
*
Llo D4968
Llo NSW150
WT Lpn
ΔT4 Lpn
Co-infection
D4968:
NSW150:
Lpn:
WT
WT

Figure S3

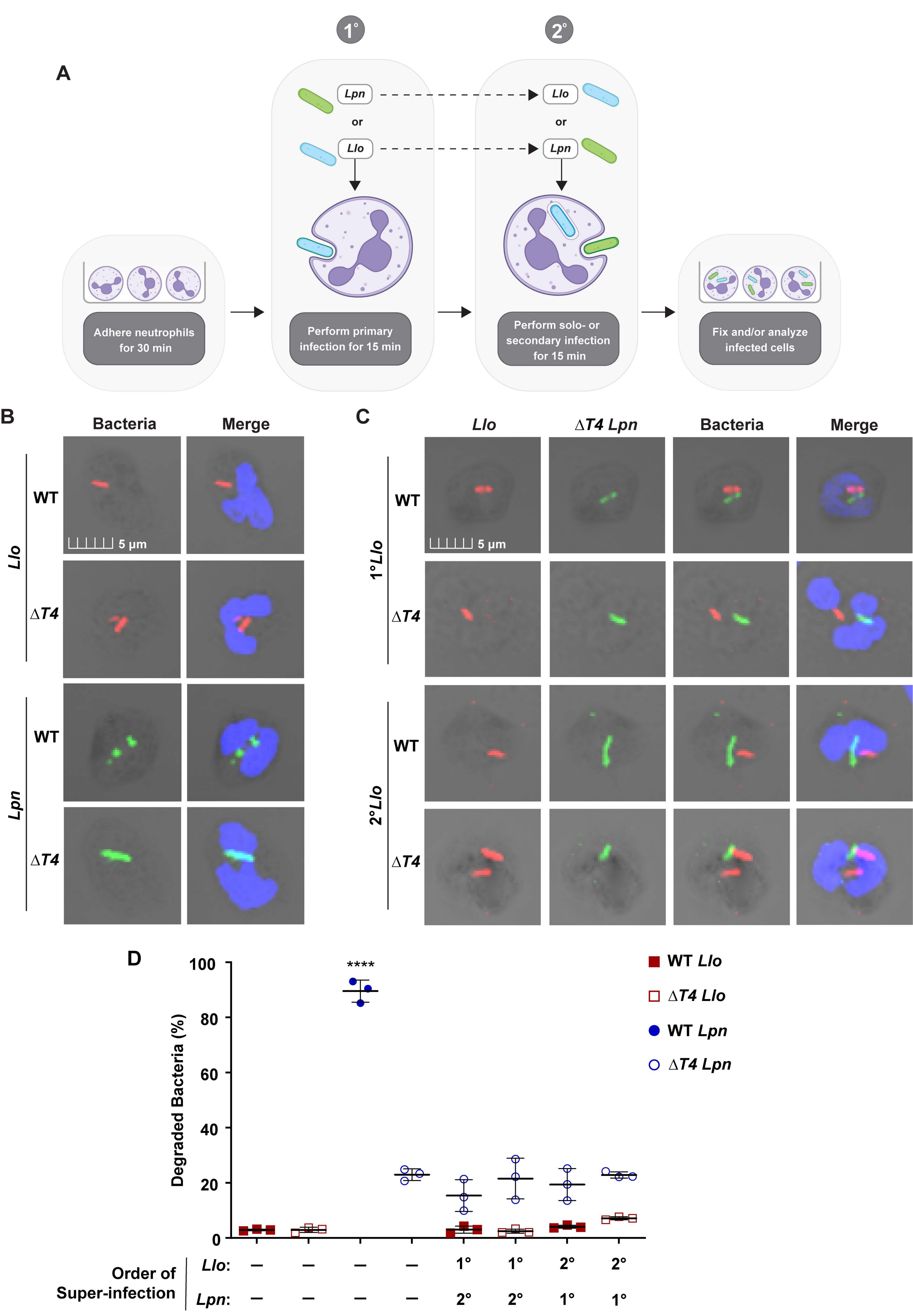

A
1°
2°
Lpn
or
Llo
Llo
or
Lpn
Adhere neutrophils for 30 min
Perform primary infection for 15 min
Perform solo- or secondary infection for 15 min
Fix and/or analyze infected cells
B
Bacteria
Merge
Llo
WT
ΔT4
Lpn
WT
ΔT4
5 µm
C
Llo
ΔT4 Lpn
Bacteria
Merge
1°Llo
WT
ΔT4
2°Llo
WT
ΔT4
5 µm
D
Degraded Bacteria (%)
100
80
60
40
20
0
****
WT Llo
ΔT4 Llo
WT Lpn
ΔT4 Lpn
Order of Super-infection
Llo: – – – – 1° 1° 2° 2°
Lpn: – – – – 2° 2° 1° 1°

Figure S4

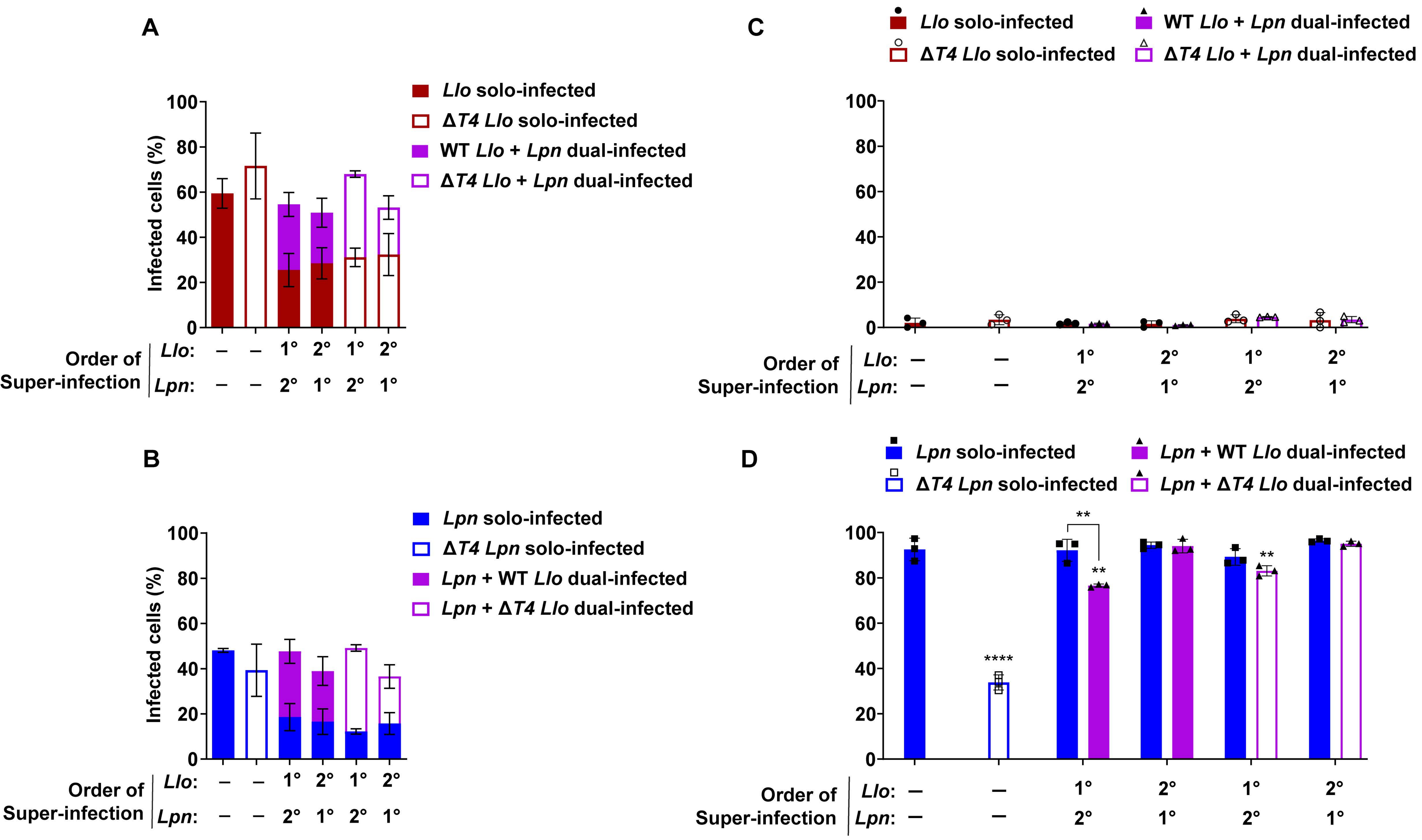
A
Llo solo-infected
ΔT4 Llo solo-infected
WT Llo + Lpn dual-infected
ΔT4 Llo + Lpn dual-infected
Infected cells (%)
0
20
40
60
80
100
Order of Super-infection
Llo: – – 1° 2° 1° 2°
Lpn: – – 2° 1° 2° 1°
B
Lpn solo-infected
ΔT4 Lpn solo-infected
Lpn + WT Llo dual-infected
Lpn + ΔT4 Llo dual-infected
Infected cells (%)
Order of Super-infection
Llo: – – 1° 2° 1° 2°
Lpn: – – 2° 1° 2° 1°
C
Llo solo-infected
WT Llo + Lpn dual-infected
ΔT4 Llo solo-infected
ΔT4 Llo + Lpn dual-infected
Order of Super-infection
Llo: – – 1° 2° 1° 2°
Lpn: – – 2° 1° 2° 1°
D
Lpn solo-infected
Lpn + WT Llo dual-infected
ΔT4 Lpn solo-infected
Lpn + ΔT4 Llo dual-infected
****
**
**
Order of Super-infection
Llo: – – 1° 2° 1° 2°
Lpn: – – 2° 1° 2° 1°

Figure S5

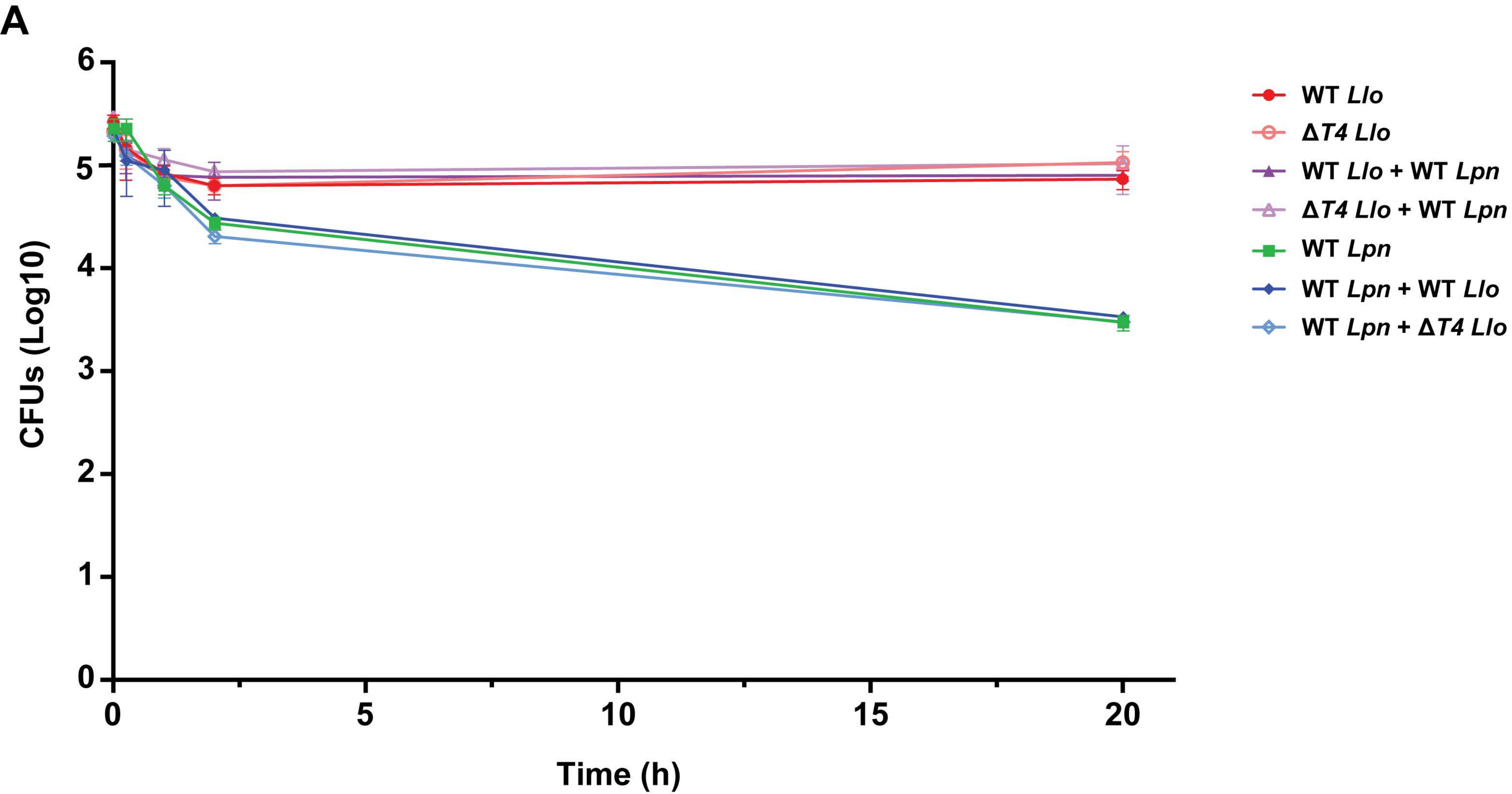
A
6
5
4
3
2
1
0
CFUs (Log10)
0
5
10
15
20
Time (h)
WT *Llo*
Δ*T4 Llo*
WT *Llo* + WT *Lpn*
Δ*T4 Llo* + WT *Lpn*
WT *Lpn*
WT *Lpn* + WT *Llo*
WT *Lpn* + Δ*T4 Llo*

Figure S6

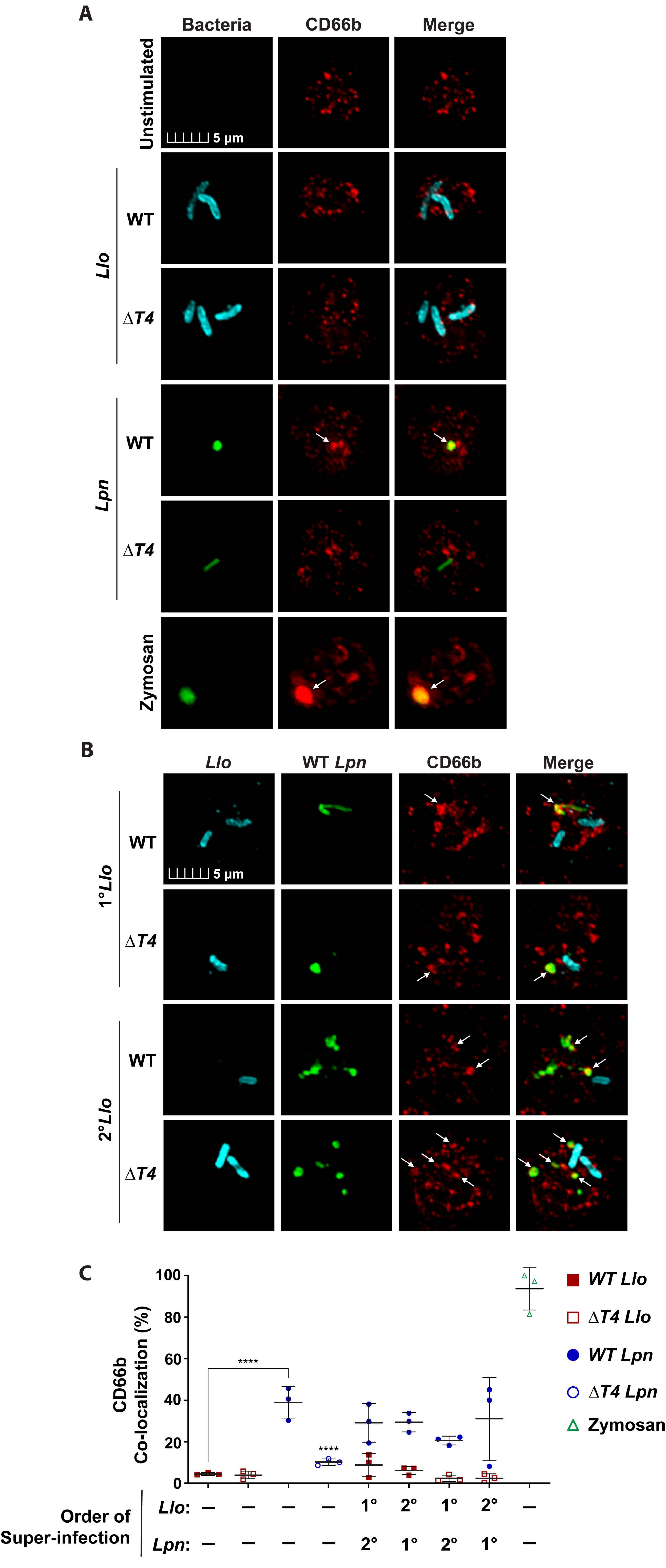
A
Bacteria
CD66b
Merge
Unstimulated
5 μm
Llo
WT
ΔT4
Lpn
WT
ΔT4
Zymosan
B
Llo
WT Lpn
CD66b
Merge
1°Llo
WT
5 μm
ΔT4
2°Llo
WT
ΔT4
C
CD66b Co-localization (%)
100
80
60
40
20
0
****
****
WT Llo
ΔT4 Llo
WT Lpn
ΔT4 Lpn
Zymosan
Order of Super-infection
Llo: – – – – 1° 2° 1° 2° –
Lpn: – – – – 2° 1° 2° 1° –

Figure S7

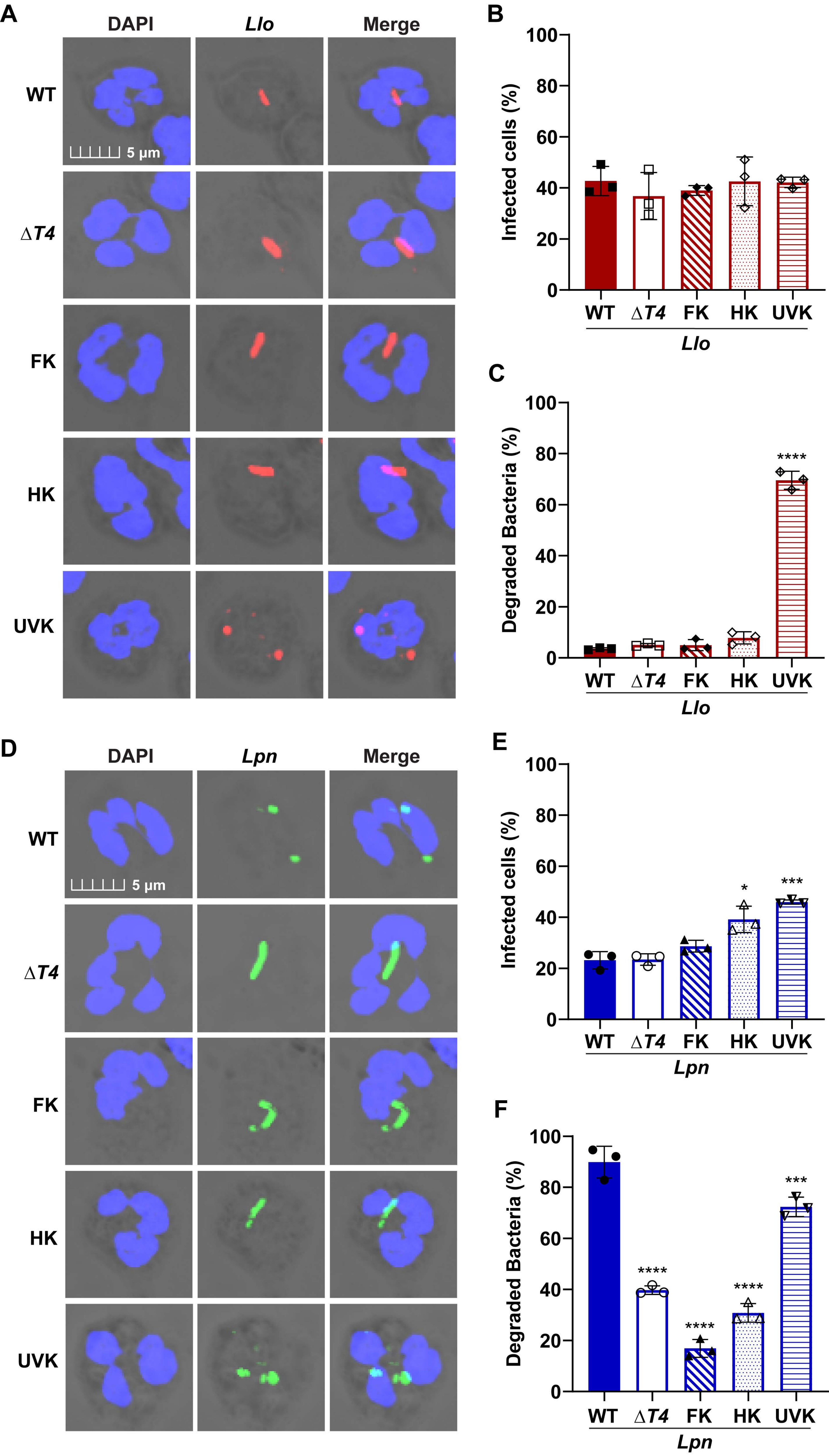
A
DAPI
Llo
Merge
WT
ΔT4
FK
HK
UVK
5 μm
B
Infected cells (%)
100
80
60
40
20
0
WT
ΔT4
FK
HK
UVK
Llo
C
Degraded Bacteria (%)
****
D
DAPI
Lpn
Merge
E
Infected cells (%)
*
***
Lpn
F
Degraded Bacteria (%)
****
***

Figure S8

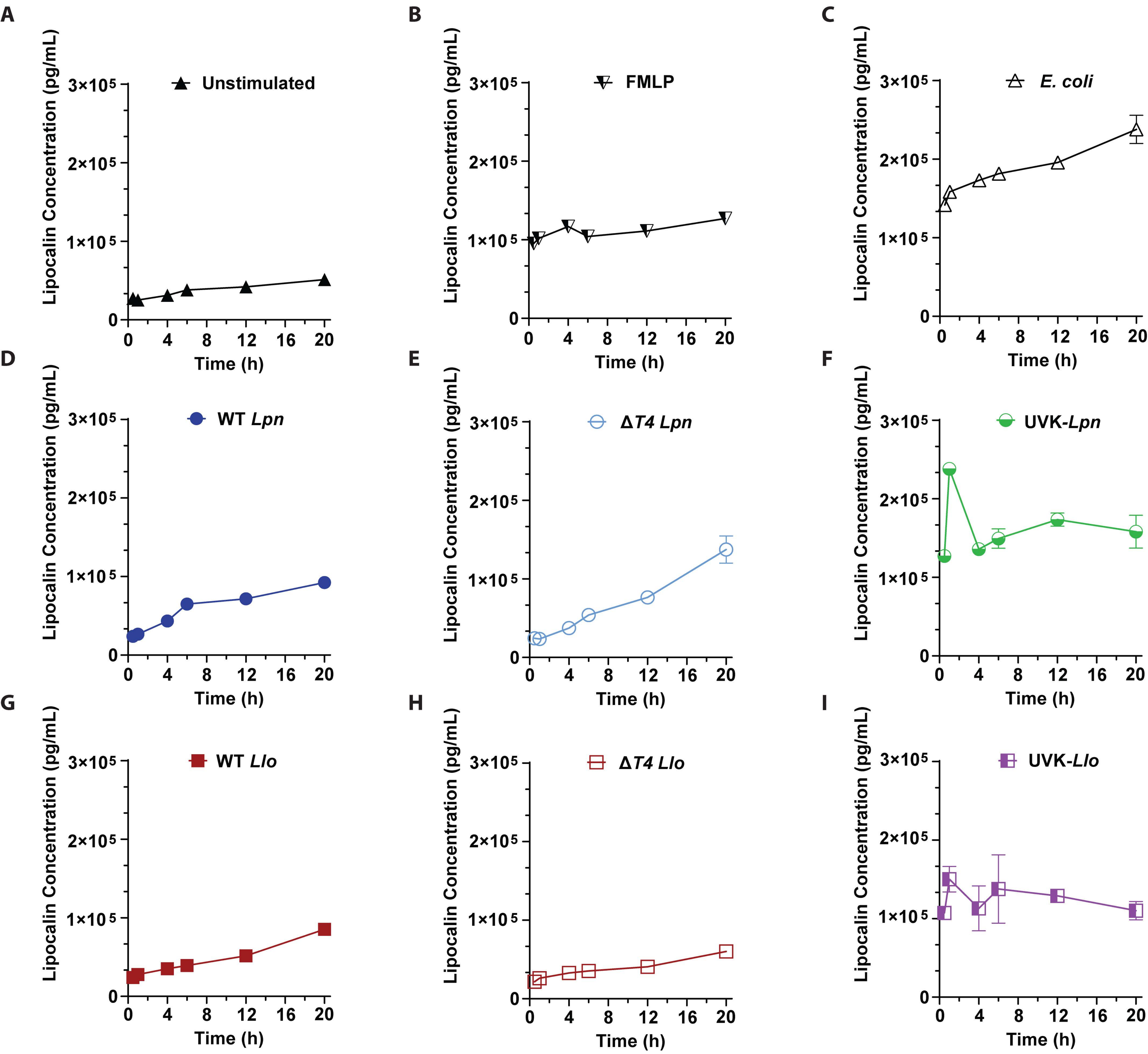
A
B
C
D
E
F
G
H
I
Unstimulated
FMLP
E. coli
WT Lpn
ΔT4 Lpn
UVK-Lpn
WT Llo
ΔT4 Llo
UVK-Llo
Lipocalin Concentration (pg/mL)
Time (h)
0
1×10⁵
2×10⁵
3×10⁵
0 4 8 12 16 20

Figure S9

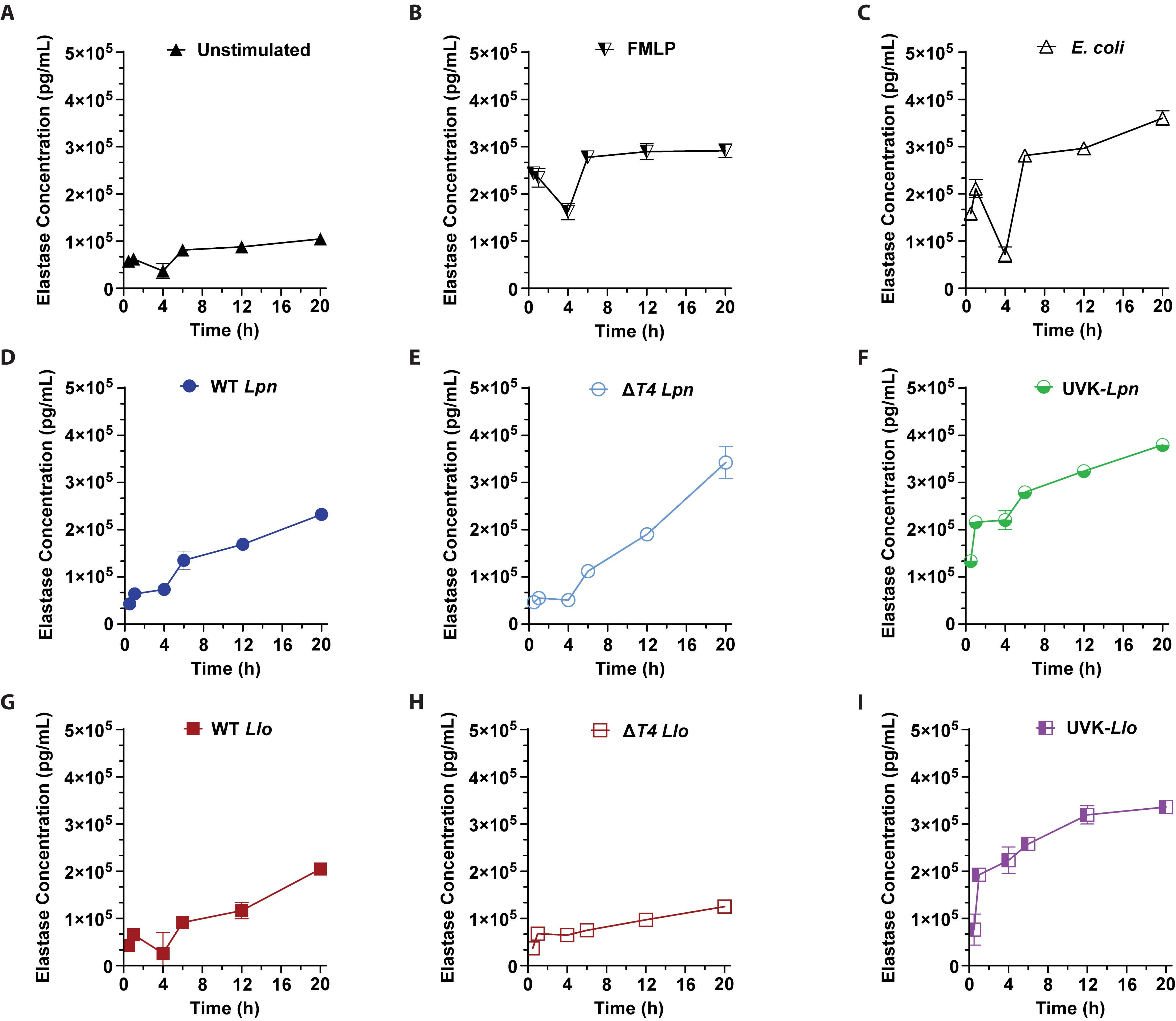
A
Unstimulated
B
FMLP
C
E. coli
D
WT Lpn
E
ΔT4 Lpn
F
UVK-Lpn
G
WT Llo
H
ΔT4 Llo
I
UVK-Llo
Elastase Concentration (pg/mL)
Time (h)
5×10^5
4×10^5
3×10^5
2×10^5
1×10^5
0
0
4
8
12
16
20

Figure S10

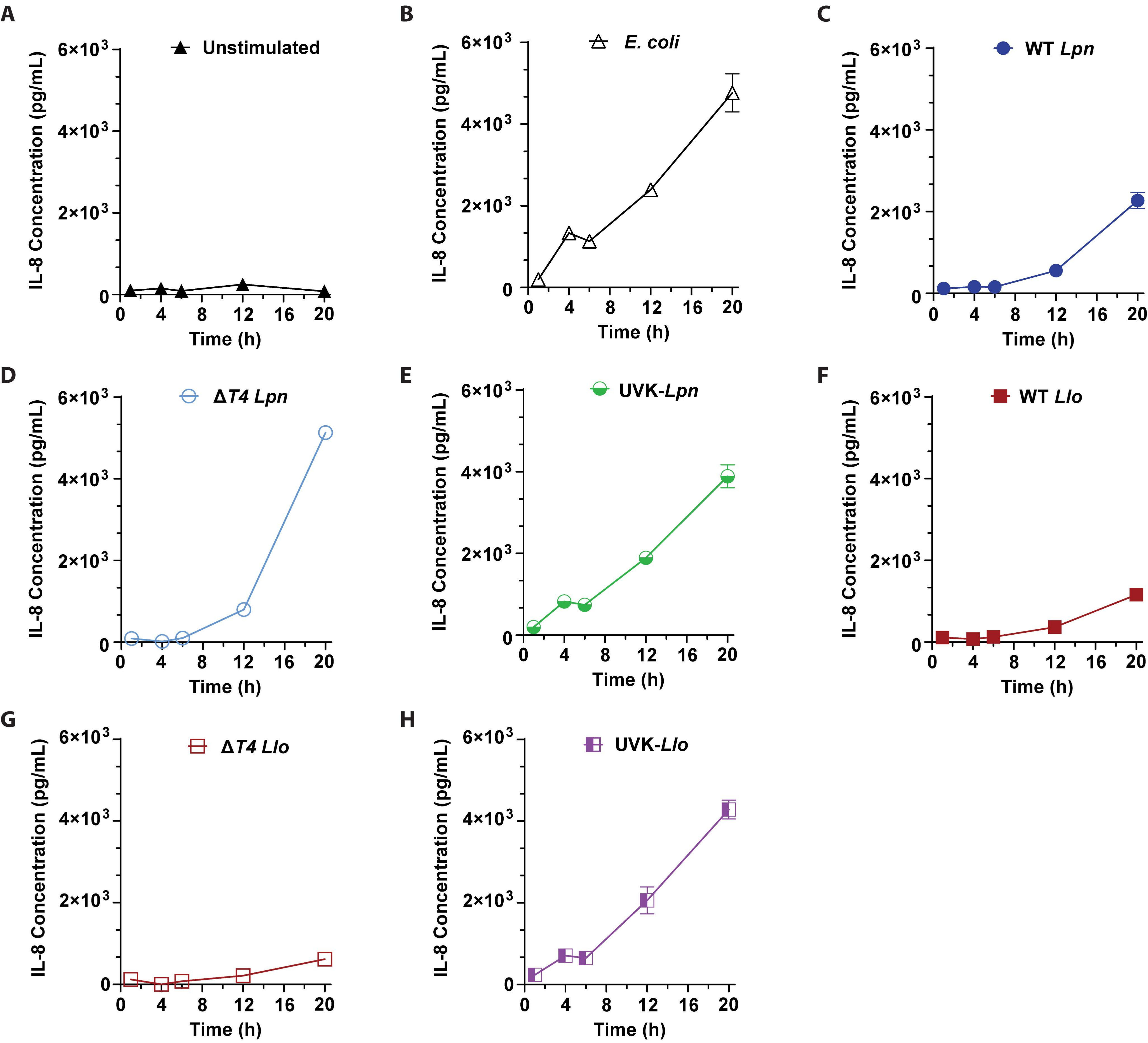
A
Unstimulated
B
E. coli
C
WT Lpn
D
ΔT4 Lpn
E
UVK-Lpn
F
WT Llo
G
ΔT4 Llo
H
UVK-Llo
IL-8 Concentration (pg/mL)
Time (h)
6×10³
4×10³
2×10³
0
0
4
8
12
16
20
